# Inherited determinants of early recurrent somatic mutations in prostate cancer

**DOI:** 10.1038/s41467-017-00046-0

**Published:** 2017-06-29

**Authors:** Alessandro Romanel, Sonia Garritano, Blerta Stringa, Mirjam Blattner, Davide Dalfovo, Dimple Chakravarty, David Soong, Kellie A. Cotter, Gianluca Petris, Priyanka Dhingra, Paola Gasperini, Anna Cereseto, Olivier Elemento, Andrea Sboner, Ekta Khurana, Alberto Inga, Mark A. Rubin, Francesca Demichelis

**Affiliations:** 1 Centre for Integrative Biology, University of Trento, Via Sommarive 9, 38123 Trento, Italy; 2 Caryl and Israel Englander Institute for Precision Medicine, New York Presbyterian Hospital–Weill Cornell Medicine, 413 East 69th Street, New York, NY 10021 USA; 3 Department of Physiology and Biophysics, Weill Cornell Medicine, 1300 York Avenue, New York, NY 10065 USA; 4 Department of Pathology and Laboratory Medicine, Weill Cornell Medicine, 1300 York Avenue, New York, NY 10065 USA; 5 Sandra and Edward Meyer Cancer Center at Weill Cornell Medicine, 1300 York Avenue, New York, NY 10065 USA

## Abstract

Prostate cancer is a highly heritable molecularly and clinically heterogeneous disease. To discover germline events involved in prostate cancer predisposition, we develop a computational approach to nominate heritable facilitators of somatic genomic events in the context of the androgen receptor signaling. Here, we use a ranking score and benign prostate transcriptomes to identify a non-coding polymorphic regulatory element at 7p14.3 that associates with DNA repair and hormone-regulated transcript levels and with an early recurrent prostate cancer-specific somatic mutation in the Speckle-Type POZ protein (SPOP) gene. The locus shows allele-specific activity that is concomitantly modulated by androgen receptor and by CCAAT/enhancer-binding protein (C/EBP) beta (CEBPB). Deletion of this locus via CRISPR-Cas9 leads to deregulation of the genes predicted to interact with the 7p14.3 locus by Hi-C chromosome conformation capture data. This study suggests that a polymorphism at 7p14.3 may predispose to SPOP mutant prostate cancer subclass through a hormone-dependent DNA damage response.

## Abstract (web summary)

Prostate cancer is a heterogeneous disease, and many cases show somatic mutations of SPOP. Here, the authors show that a non-coding polymorphic regulatory element at 7p14.3 may predispose to SPOP mutant prostate cancer subclass through a hormone dependent DNA damage response.

## Introduction

Prostate cancer (PCa) is the second most frequent cancer in men causing each year more than 250,000 deaths worldwide. From a genomic perspective PCa is a collection of molecular subclasses^1^. Approximately 58% of risk for prostate cancer has been estimated to be due to inherited genetic factors^2^. Genome-wide association studies have identified more than 100 common single-nucleotide polymorphisms (SNPs) associated with the risk of developing PCa^3^. Most of these variants reside in non-coding regulatory regions and may affect the transcription factors (TFs)-binding affinity^4^. Androgen receptor (AR) regulates genes expression in multiple tissues and diseases, by targeting binding elements in promoters and distant enhancers. A recent PCa whole-genome sequencing study revealed a significant correspondence between DNA breakpoints and AR-binding sites implicating an inter-play between hormone regulation and genomic events^5^. These studies highlight an important role of androgens in the initiation and development of PCa. Indeed, at the earliest time point of clinical presentation, PCa already harbors a range of genomic lesions^1^ possibly due to DNA repair defects. We reasoned that, over a man’s lifetime, heritable variants could potentially predispose to genomic instability in the context of variable AR signaling leading to early PCa-specific somatic genomic events. To test this hypothesis, we interrogated the constellation of transcriptomic changes in benign prostate cells for clues as to how genetic variants could impact prostate cancer development through alterations in the expression of DNA repair genes and hormone-regulated genes. Here we report a link between an inherited non-coding variant and prostate cancer somatic mutations through the interrogation of large cohorts of human data and experimental support of the functional activity of the variant locus.

## Results

### In silico selection of germline triggers of somatic mutations

**Fig. 1:**
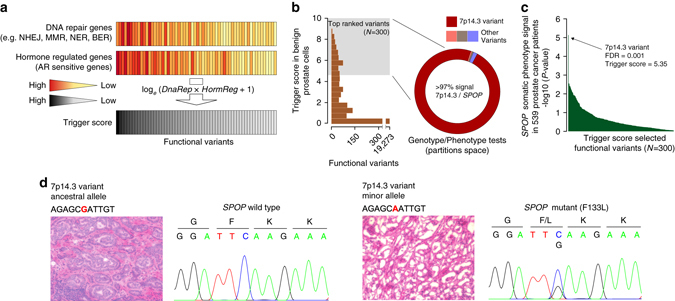
Genetic predisposition to *SPOP* mutant prostate cancer. **a** Schematic representation of the trigger score computation. The number of DNA repair (*DnaRep*) and hormone-regulated genes (*HormReg*) from healthy prostate cells that are modulated by a functional variant are combined into a ranking score that measures the likelihood to observe a prostate-specific early somatic event. The combination of the two variables demonstrate the nontrivial impact that DNA repair and hormone-regulated genes have on trigger score ranking. **b** Trigger score distribution (*left*) across all considered functional variants; top ranked variants are highlighted. Genotype/phenotype analysis (*right*) is performed on random partitions of the data set into discovery and validation sets for three early recurrent prostate cancer lesions (*SPOP* mutations, *FOXA1* mutations, and *TMPRSS2-ERG* rearrangement). An 7p14.3 variant associated to SPOP was implicated in 97.4% of all collected associations (187 of the 192 partitions for which association signal was detected, *red portion* of the ring plot). No variants in the partition space for *FOXA1* and *TMPRSS2-ERG* lesions were identified. **c** Genotype/*SPOP* phenotype data on the whole study set is shown (7p14.3 variant highlighted, dominant test considered). **d** Hematoxylin and eosin stained prostate cancer frozen tissue sections and corresponding *SPOP* Sanger sequencing are shown for a patient carrying the 7p14.3 variant ancestral genotype and lacking *SPOP* mutation (*left*) and a patient carrying the 7p14.3 variant minor allele genotype and harboring *SPOP* F133L mutation (*right*)

To quantitatively assess the predisposition to genomic changes in the context of AR signaling, we developed an approach to nominate potential heritable facilitators (referred hereafter as triggers) of somatic genomic events. We considered human variants within functionally active regions of the genome defined by the Encyclopedia of DNA Elements (ENCODE) histone mark ChIP-seq data^6^, and established a ranking score, the trigger score, which quantifies the fraction of the transcriptome putatively modulated by each human variant leveraging individuals’ genotypes and transcript levels (Fig. 1a). The trigger score-unlike eQTL-based approach-only queries a predefined set of transcripts and ranks the variants for their likelihood to play a role in predisposition to cancer hallmarks^7^. When applied to a RNA-seq data set comprising more than 200 samples including benign human prostate tissue from The Cancer Genome Atlas (TCGA) and samples from the 1000 Genomes Project with known genotype at variants in transcriptionally active regulatory elements^4, 6, 8^, the trigger score nominated 300 polymorphisms linked to DNA repair and hormone-regulated genes (Fig. 1b, Supplementary Data 1–3). Sixty-nine of those sites had a minimal trigger score in non-prostate samples (Supplementary Data 3).

Several recent genomic studies now establish PCa as best being regarded as a collection of molecularly defined cancers -similar to breast and lung cancer- with major subclasses defined by either *ETS* gene fusions (most commonly *TMPRSS2-ERG* rearrangements), *SPOP* or *FOXA1* single-nucleotide mutations^1, 9, 10^. These genomic events are recognized as early clonal events that are recurrent in primary untreated prostate cancers^9, 11^, and are mainly prostate specific. To explore genotype/phenotype relationship for these common prostate cancer mutations, we assembled a data set comprising 539 prostate tumors from three recent studies^1, 9, 11^, and observed 47.2, 12.1, and 5.4% incidence, respectively, (Supplementary Data 4). To test the relationship between the trigger candidates and the three somatic phenotypes, we used a computational in-silico cross-validation strategy that limits false positives results and implements multiple discovery and validation partitions from the entire cohort preserving somatic event incidence. No signal was detected for the *FOXA1* phenotype and, surprisingly, no signal was observed for the largest genomic subclass defined by the *ETS* rearrangement phenotype (i.e., *TMPRSS2*-*ERG*). In contrast, a polymorphic site on 7p14.3 (rs1376350) was significantly associated with *SPOP* somatic mutations in 97.4% of the partitions with any positive signal (Fig. 1b, Supplementary Data 5 and Supplementary Table 1). When interrogating the whole prostate cancer cohort (*N* = 539 patients, Fig. 1c and Table 1), the association with the phenotype was highly significant (*P* = 6.7e-06, OR = 4.83 logistic regression analysis). Further analyses including *SPOP* wild-type samples of the prostate cancer Tyrol PSA screening cohort^12^ and of the 1000 Genomes Project confirmed the association (*P* = 1.22e-08, OR = 5.54 logistic regression analysis). Moreover, the genotype/phenotype relationship with *SPOP* mutation was confirmed in an independent non-Caucasian prostate cancer cohort of Korean men (*P* = 4e-02, OR = 5.84 logistic regression analysis)^13^, indicating that this phenomenon is not limited to Caucasian male populations but rather may extend across multi-ethnic populations (Table 1 and Fig. 1d). No association was detected between the genotype and the total number of somatic single-nucleotide variants (SNVs) in the tumor, but we observed increased somatic genomic burden in men with the minor allele associated with *SPOP* mutant prostate cancer (Supplementary Fig. 1). Linkage disequilibrium analysis on the 1000 Genomes Project data did not reveal variants in functional or coding regions linked to rs1376350 across populations (Supplementary Data 6).

**Table 1:** Association signal of variant 7p14.3 with SPOP somatic phenotype

Cohort	AA+AG carriers	GG carriers	MAF	Comparison with SPOP.wt tumors		Comparison with tyrol controls (*N* = 1,014)		Comparison with tyrol extended controls # (*N* = 1,291)		Comparison with 1000 Genomes Project controls ## (*N* = 2,504)		Comparison with all controls ## (*N* = 3,795)	
				OR	*P*	OR	*P*	OR	*P*	OR	*P*	OR	*P*
Adenocarcinomas discovery	24	217	0.052	5.75	3.0e-04	10.47	1.1e-07	10.2	9.1e-08	5.44	3.0e-05	6.38	4.5e-06
Adenocarcinomas validation	23	217	0.048	4.45	4.1e-03	8.22	8.8e-06	7.9	9.1e-06	4.04	1.5e-03	4.73	4.0e-04
Adenocarcinomas complete	47	434	0.050	4.83	6.7e-06	9.20	1.54e-10	8.9	7.4e-11	4.72	3.0e-07	5.54	1.2e-08
Adenocarcinomas EUR only	42	373	0.052	4.96	3.7e-05	10.07	1.3e-09	9.75	7.4e-10	7.86	1.1e-07**	8.44	7.8e-10**
Validation Korean	19	61	0.206	5.84	4.0e-02	–	–	–	–	4.78	4.3e-02***	–	–

Results refer to logistic regression analysis using dominant model corrected for age and prostate-specific antigen (*PSA*). First three rows show data from a random partition (discovery and validation) and the complete data set; columns include signal upon data set extension to controls from the Tyrol PSA Screening Cohort and the 1000 Genomes Project individuals collection. Data is also reported for EUR descent individuals only and for an independent cohort of Korean patients (EAS from 1000 Genomes Project collection included as controls). #ETS positive/SPOP.wt tumors and controls, ##analysis not corrected for age and PSA, **EUR individuals only included (*N* = 503), ***EAS individuals only included (*N* = 504).

### Regulatory impact of 7p14.3 variant

Fourteen DNA repair genes and 15 hormone-regulated genes contributed to the high trigger score for the 7p14.3 variant (Supplementary Data 7), of which *DAZAP2*, *DDX18*, *SET*, and *XRCC5* were also significantly deregulated in *SPOP* mutant as compared to *SPOP* wild-type human prostate carcinoma cases (Supplementary Fig. 2). Interestingly, 93% of DNA repair and hormone-regulated transcripts are downregulated by the 7p14.3 variant minor allele; this proportion is significantly higher than the fraction of transcripts downregulated by the 7p14.3 minor allele in the whole transcriptome (48%, *P* = 3.2e-06 proportion test). Protein–protein-interaction (PPI) network data projected on the fraction of the whole transcriptome modulated by the variant (Supplementary Fig. 3) revealed a connected subnetwork of significant relative size when compared to the same analysis for each polymorphic transcriptionally active regulatory element considered in the study (*P* = 1e-02 resampling, Supplementary Fig. 4). Genes in the subnetwork are significantly enriched for gene expression and translation pathways terms (FDR = 5.1e-17 and FDR = 1.6e-07, respectively) and, as expected, for hormone-regulated and DNA repair genes (*P* = 0.048, *P* = 6e-04 permutation test). In addition, of all the 57 oncogenes^14^ including 39 TFs we queried for targets enrichment^15^, c-MYC resulted as the only significant one (FDR = 0.005; *P* < 1e-05 permutation test) with a majority of downregulated targets (*P* = 0.018 proportion test). Overall, this suggests a broad transcriptional regulatory impact of the variant 7p14.3^4^.

### In vitro validation

**Fig. 2:**
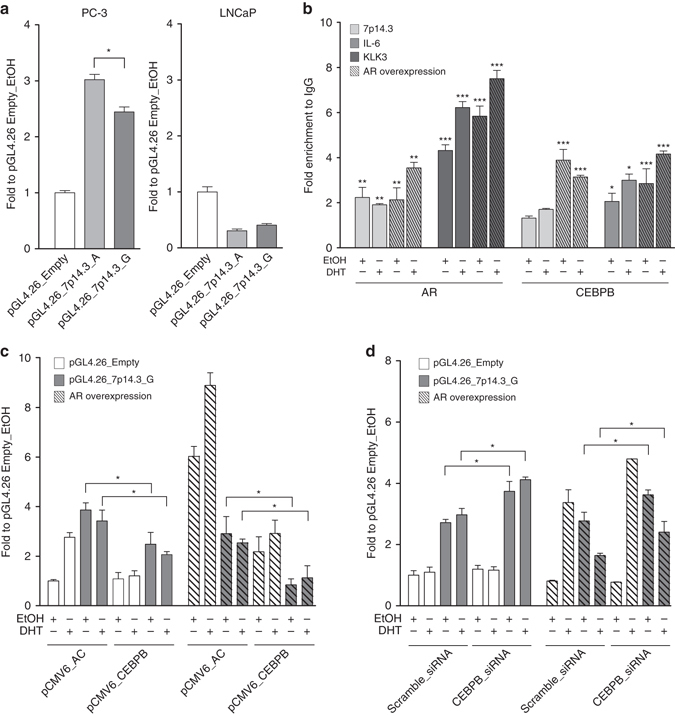
Functional characterization of 7p14.3 variant. **a** Luciferase assays were performed on PC-3 and LNCaP cells transfected with pGL4.26 vectors containing 7p14.3 (A or G allele, represented in *light grey* and *dark grey*, respectively) or empty vector (*white*); mean ± s.d. of three biological replicates. **b** PC-3 cells were transfected with pCMV_Empty (*solid bars*) or pCMV_AR (*dashed bars*) vectors; AR (*left*) or CEBPB (*right*) chromatin binding at 7p14.3 locus in PC-3 cells were evaluated by ChIP-qPCR. Occupancy level at *KLK3* enhancer and *IL-6* promoter was used as positive control of AR and CEBPB, respectively. Data are represented as mean ± s.d. of two biological replicates. **c** Luciferase assays on PC-3 cells co-transfected with pCMV6_CEBPB and/or CMV_AR (*dashed bars*) along with the different pGL4.26 reporter vectors described above. The enhancer activity is inhibited upon CEBPB overexpression. The inhibition becomes stronger upon AR over-expression. Data are represented as mean ± s.d. of two biological replicates. **d** Luciferase assays on PC-3 cells transfected with siRNA against CEBPB or scrambled siRNA. Then, cells were co-transfected with pCMV_Empty or pCMV_AR vectors along with the pGL4.26 reporter vectors described above. Data are represented as mean ± s.d. of two biological replicates. Where indicated, cells were treated for 16 h with EtOH or DHT. *P < 0.05,.**P < 0.01, ***P < 0.005, Student’s *t*-test

We then verified the activity of the polymorphic regulatory region containing the 7p14.3 variant with an in vitro luciferase assay in two model systems, AR-negative (PC-3) and AR-positive (LNCaP) prostate cancer cells (Fig. 2a). In PC-3 cells, significantly increased activity was observed in the presence of the minor allele (adenine) associated with *SPOP* mutation compared to the ancestral one (guanine). In contrast, inhibitory activity was observed in LNCaP cells, suggesting differential effects of the variant with respect to AR status. TF DNA-binding site (TFBS) motifs analysis demonstrated an AR consensus motif at the variant locus with the minor but not with the ancestral allele (Supplementary Fig. 5a, Supplementary Data 8). In addition, we identified a consensus motif for the CEBP family (Supplementary Fig. 5b), which includes known AR co-repressors^16^. RNA-seq data show high levels of CEBPB transcripts in multiple prostate tissue cell lines and a marked anti-correlation with AR levels in human prostate cancers (*N* = 319, *P* = 8e-18 Pearson correlation, Supplementary Fig. 6a, b). A less stringent TFBS search in a wider genomic region revealed additional CEBPB-specific consensus motifs in proximity of the variant locus. In addition, we found overlapping CEBPB and AR motifs ~70 bp downstream the variant and a CEBPB putative-binding site ~180 bp upstream the variant, along with motifs for MAFB and c-MYC TFs, known to co-localize with CEBPB^17^ (Supplementary Data 8). We, therefore, investigated the effect of AR over-expression on TFs binding at the 7p14.3 locus.

ChIP-qPCR experiments showed AR and CEBPB recruitment at the site of interest upon AR over-expression (KLK3 and IL-6 were used as positive controls, respectively) (Fig. 2b). Based on the wider TFBS search results, we also verified the recruitment of c-MYC at the 7p14.3 region upon AR over-expression and dihydrotestosterone (DHT) treatment (Supplementary Fig. 7). To address the potential functional consequences of AR and CEBPB binding to the polymorphic site, we examined the effects of modulating androgenic signaling. We observed decreased responsiveness of the reporter constructs in PC-3 cells upon AR overexpression and DHT treatment (Supplementary Fig. 8), whereas loss of repressive activity upon AR knock-down in LNCaP cells was seen (Supplementary Fig. 9a, b). PC-3 cells also showed a decreased enhancer activity upon CEBPB over-expression, which was even stronger upon AR over-expression (Fig. 2c), and an increase in enhancer transactivation upon CEBPB knock-down (Fig. 2d). The construct harboring the minor allele (A) showed the same behavior, while eliciting higher enhancer activity at all conditions (Supplementary Fig. 10). These data suggest that CEBPB may act as an AR co-repressor at the variant locus, potentially through its recruitment by AR and that the 7p14.3 locus may undergo allele-dependent TF binding altering the expression of DNA repair and hormone-dependent genes.

### Structural associations with 7p14.3 locus

**Fig. 3:**
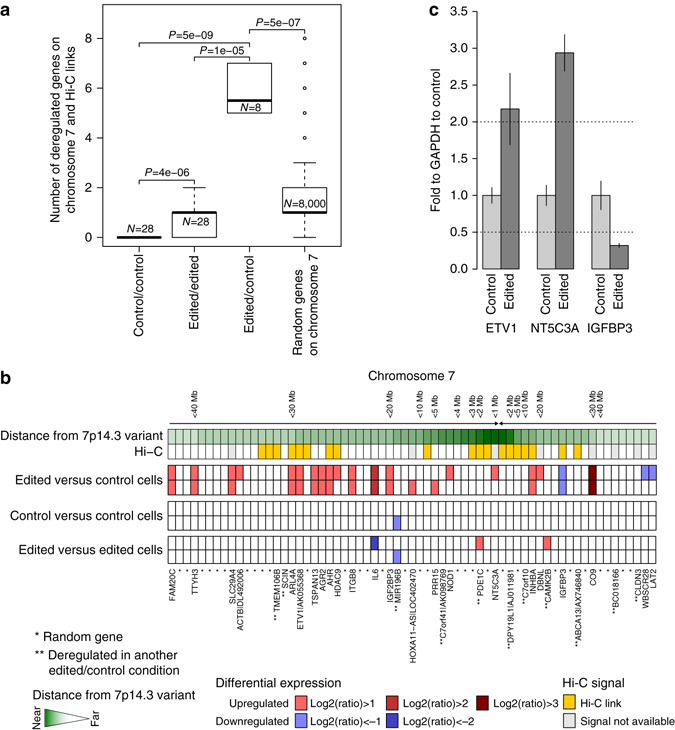
Transcriptome of 7p14.3 locus deleted cells. **a** Deregulation of transcripts on chromosome 7 with respect to prostate cells Hi-C identified links. Enrichment is shown by comparing the level of deregulation in edited vs. control cells, in edited vs. edited, and in control vs. control cells. Further, enrichment is shown by comparing the level of deregulation in edited vs. control cells with simulated data computed by generating, for each tested combination, 1000 random selections of genes at chromosome 7 with size equal to the observed deregulated set. *P*-values are computed using Mann–Whitney test. **b** Visual representation of deregulation patterns in edited vs. control cells at chromosome 7 within a 40 Mb window around the 7p14.3 variant. Representative experimental conditions of edited vs. control cells are shown and random combinations of edited vs. edited and control vs. control cells are shown. **c** Real-time PCR validation of selected genes nominated by RNA-seq, ETV1, NT5C3A, and IGFBP3; *dotted lines* represent fold thresholds applied in RNA-seq analysis to identify deregulated transcripts (additional data and negative controls in Supplementary Fig. 12). Data are represented as mean ± s.d. of three technical replicates

To further demonstrate that the 7p14.3 region is functionally active, we deleted 731 bp of genomic sequence around the site via CRISPR-Cas9 (Supplementary Data 9) in PC-3 cells and sequenced their transcriptomes. Differential transcript expression analysis of edited vs. non-edited cells (control cells) showed enriched deregulation when compared to the same analysis in edited vs. edited and control vs. control (*P* = 1e-04 and *P* = 3e-05 Mann–Whitney test, respectively, Supplementary Fig. 11) as validated by real-time PCR in selected genes (Supplementary Fig. 12). The fraction of deregulated genes that showed upregulation resulted on average in 70%, 65%, and 62%, respectively. Along chromosome 7 the analysis between edited vs. control cells, but not edited vs. edited or control vs. control, showed significant concordance with genes predicted to physically interact with the 7p14.3 locus by previously generated Hi-C chromosome conformation capture data from benign prostate cells^18^ (Fig. 3a, b, *P* = 1e-05 and *P* = 5e-09 Mann–Whitney test, respectively). Validation of *ETV1*, *NT5C3A* and *IGFBP3* deregulation upon deletion of 7p14.3 locus is shown (Fig. 3b, c) further supporting medium-range interaction.

## Discussion

Over the past 15 years, numerous non-coding SNPs have been linked to the susceptibility to developing prostate cancer^3^, the second most frequent cancer in men causing each year more than 250,000 deaths worldwide, with modest albeit highly significant effects. Evidence of predisposition to *TMPRSS2-ERG* prostate cancer subtype was previously tested in small familial or sporadic small cohorts^19–21^. Recently, two out of 27 common prostate cancer risk variants were found associated with modest signal to the ETS subclass^22^. Here we tested a specific hypothesis on large collections of human prostate tissues and identified a strong association with an emerging class involving mutation in the *SPOP* gene. Its phenotype is related to DNA repair and AR dysfunction^11, 23^ and defines a distinct molecular class as confirmed by the TCGA prostate adenocarcinoma publication^1^. Our findings suggest that the genetic component of this common disease is linked, at least partially, to specific molecularly defined sub-classes through the modulation of AR targets and DNA repair genes. *SPOP* mutations that predominately involve hotspots located in the MATH substrate binding domain of the protein are prostate cancer specific^24^. Using in vitro models, we recently linked the *SPOP* mutant prostate cancer to genomic instability due to defects in homologous repair^23^.

**Fig. 4:**
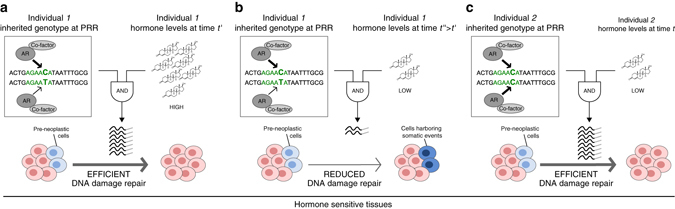
Two-variable model of genotype–environment interaction study. Three combinations of individual’s genotypes (ancestral allele, cytosine) at a polymorphic regulatory region (PRR) and hormone levels (high, low) are represented as examples of the study rationale. **a** Heterozygous genotype of Individual 1 and high hormone levels maintain DNA damage repair efficiency; **b** in the presence of low hormone levels for the same individual, reduced transcription of DNA repair genes is expected, facilitating the emergence of early somatic events; **c** low hormone levels do not impair DNA damage repair efficiency of Individual 2 who inherited ancestral homozygous genotype at the site. Within sequences, *green* nucleotides indicate AR half motif; bold identifies the SNP locus in the regulatory region [C/T]. The described interaction is not relevant to hormone insensitive tissues. Based on the specific study results, we postulate that in case of the rs1376350 locus AR mediates repression activity partially through the negative regulation of CEBPB and its recruitment to the polymorphic regulatory locus where the minor allele demonstrates higher AR affinity

While the mechanism linking the 7p14.3 variant and *SPOP* mutation remains elusive and future studies should investigate the role of the allele in the emergence of *SPOP* somatic mutations, we propose a relevant role in cancer predisposition of non-coding variants that lead to allele-specific transactivation of central TF programs with age dependent and tissue-specific effect^8, 21, 25^ manifesting in early somatic genomic events. This study has potential important implications for the aging male whose testosterone levels change with advancing age, where a subtle differential effect might become significant to the cell (Fig. 4, Supplementary Fig. 13) and facilitate or accelerate the initiation of tumorigenesis in hormone sensitive tissues.

Findings from this study may be generalizable to other cancer subtypes. The predisposition to neoplastic development manifests in a combination of genomic and epigenetic events over time. Unlike many of the model systems that require results to occur in short periods of time, neoplastic development is a fitness selection process. As suggested with the 7p14.3 allele in this study, association with disease-specific pathways potentially enables a cascade of events over time to provide an advantage for cells that are hormonally regulated, for example, to undergo more frequent DNA damaging events that are not repaired. As in the current study, this genetic predisposition may positively select for *SPOP* mutations as they accelerate the DNA damage phenotype with a complex interplay of multiple TFs at the site of interest involving AR, CEBPB, and possibly c-MYC. Future large scale studies should explore the role of germline trigger events to announce driver somatic mutations.

## Methods

### Selection of human genome variants in functional regions

ChIP-Seq ENCODE data were queried for 16 cell-lines selected based on availability of H3K4m1, H3K4m3, and H3K27ac regions of signal enrichment data (broadPeak format). The cell line set includes GM12878, H1-hESC, Hela-S3, HepG2, HMEC, HSMM, HSMMtube, HUVEC, K562, Monocytes-CD14 + , NHA, NHDF-Ad, NHEK, NHLF, Osteobl, Dnd41. Consensus regions were determined for all three marker signals. Specifically, for each marker the consensus was generated as the overlap of all regions with enrichment signal above five detected in at least two cell lines as follow: (i) retain a region if it overlaps at least another cell line region for at least 50% of its length; (ii) concatenate the coordinates of regions from step i; (iii) sort and merge retained regions. BEDTools^26^ and ENCODE data as of January 2014 were used. Next we considered known SNVs that were commonly genotyped in prostate tumors and matched healthy DNA that demonstrated high quality hybridization signal^9, 12^ and the interim TCGA prostate cancer cohort^1^. Variants within functional regions supported by presence of H3K4me1 and H3K27ac consensus signals and absence of H3K4me3 consensus signal were selected retaining only one among multiple completely dependent variants (linkage disequilibrium data provided by the International HapMap Project (http://hapmap.ncbi.nlm.nih.gov/) for all populations was used; we define two variants to be completely dependent when both *D’* and *R* ^2^ measures are equal to 1). A total of 21,364 variants in functional regions were finally considered (hereafter referred to as ‘‘functional variants’’).

### Selection of DNA repair genes and hormone-regulated genes

DNA repair genes list was obtained from the Human DNA Repair Genes database (http://sciencepark.mdanderson.org/labs/wood/dna_repair_genes.html) and an additional curated list (Supplementary Data 1) (*N* = 180). The list of hormone-regulated genes was obtained from^27^ (*N* = 330). Specifically, data for three biological replicates of LNCaP cells treated with small interfering RNA (siRNA) targeting AR and corresponding controls were considered (GSM288299, GSM288300, GSM288301, GSM288293, GSM288294, GSM288295) and for each replicate differentially expressed genes were selected as follows: (i) selection and quintile normalization of genes with reported detection *P*-values < 5% both in AR treated and control cells; (ii) Selection of genes with absolute-change, i.e., log2(treated/control), equal or greater than 1. The final hormone-regulated gene list (Supplementary Data 2) is obtained by merging the genes differentially expressed in at least one of the three replicates.

### Somatic phenotype data sets

Whole-exome or whole-genome sequencing data from prostate cancer tissue samples was queried for early somatic lesions^1, 9, 11^. Patients with relevant clinical annotations (age, PSA), functional variant genotypes and lesion status for *SPOP* (*N* = 539, 12.1% mutated), *TMPRSS2-ERG* (*N* = 451, 47.2% rearranged) and *FOXA1* (*N* = 520, 5.4% mutated) were included in the study (*N* total = 539, Supplementary Data 4). Variants genotypes were determined using standard APT tools 1.16.1 pipeline from Affymetrix SNP 6.0. As all data sets used clinically localized prostate cancer cases, none of these data sets have meaningful clinical follow up data, which would require ten or more years.

### Ethnicity analysis

Ethnicity of all individual’s samples was inferred using an approach based on inspection of differential germline variants genotype. First, by combining genotype data of individuals with known ethnicity a reference model is built; genotype data by the International HapMap Project was used. A target model is then created using genotype data from all 539 individuals in the somatic data set. Principal component analysis (PCA) is then performed by means of smart pca module^28^ on aggregated target and reference models genotype data. Euclidean space defined by the first two PCA components is then inspected to, first, generate smallest convex sets identifying main ethnic groups (EUR, AFR, EAS, AMR, and SAS) and then to annotate the ethnicity of the 539 individuals in the somatic data set. Individuals within an ethnic group set are annotated with the corresponding ethnicity; individuals outside the ethnic group sets are annotated with the nearest (Euclidean distance) ethnic group. The annotation of ethnic background in our cohort is reported in Supplementary Data 4.

### Korean cohort

Prostate cancer patients of Korean descent were previously annotated for *SPOP* mutations^13^. Individuals with relevant clinical annotations (age and PSA) and *SPOP* mutation status (*N* = 80, 8.7% *SPOP* mutated) were retained. The rs1376350 variant genotype was assessed by TaqMan assay (Supplementary Data 4).

### Control cohorts

Genotype data for the rs1376350 study variant was retrieved for 2504 individuals from the 1000 Genomes Project FTP repository (Release 20130502). Genotype data and clinical information for 1903 individuals from the Tyrol Early Prostate Cancer Detection Program cohort^12, 29^ were queried. This set includes 1036 healthy controls and additional 492 individuals considered as *SPOP* wild type (Supplementary Data 4). No statistically significant genotype/phenotype association was found when testing the 7p14.3 variant in the Tyrol cohort against prostate cancer risk (*P* = 0.47, logistic regression analysis), *TMPRSS2-ERG* rearrangement (*P* = 0.11, logistic regression analysis) or aggressive PCa (*P* = 1, logistic regression analysis).

### Transcriptome analysis and trigger score assessment

Benign (*N* = 63) and tumor (*N* = 319) prostate tissues RNA-seq data with available FASTA files^1, 9, 30^ and matched genotype data were aligned to the reference genome hg19 using STAR aligner^31^ and logarithm transformed (two based) RPKM+1 of each gene (UCSC knownGenes) were computed using mrfQuantifier^32^ and were quintile normalized.

For each functional variant, using matched normal RNA and genotype data, the fraction of modulated DNA repair and hormone-regulated genes was quantified from 459 sequenced transcripts (normalized RPKM greater or equal to 1 in at least one individual was required). Seven-hundred eighty-seven variants with monomorphic genotype in the benign samples set were excluded. Linear regression of RPKMs across genotype classes, also grouped based on dominant model or recessive model (dosage, dominant, or recessive test) was applied. Three genotype classes were required to apply the dosage test and minimum of 3% per class for dominant and recessive. For each variant, the percentage of DNA repair and hormone-regulated genes was computed as the highest percentage of associated transcripts applying a false discovery rate (FDR) threshold of 5% and the corresponding number of associated DNA repair and hormone-regulated genes were then combined to compute the trigger score defined as follows:

$${\log _e}\left( {Dna{\it{Rep}} \times {\it{HormReg}} + 1} \right)$$

Among the 21,364 functional variants considered in the study only 881 (~4%) had a positive trigger score (Supplementary Data 3). Top ranked variants in the highest tertile of positive trigger score distribution were retained for further analysis (*N* = 300). The relationship between the variants minor allele frequencies (MAF) and trigger scores was investigated (Supplementary Fig. 14); no association detected.

For the 7p14.3 variant we then also performed genome-wide association analysis considering 18,758 sequenced transcripts (all transcripts with normalized RPKM greater or equal to 1 in at least one individual). Linear regression of RPKMs across genotype classes using dosage model was performed applying a FDR threshold of 5%. The variant was found genome-wide associated with other 1515 genes of which 723 (48%) show downregulation in presence of the minor allele while 792 (52%) present upregulation.

DNA repair and hormone-regulated genes associated with 7p14.3 variant (Supplementary Data 7) were tested for differential expression across *SPOP* mutant and *SPOP* wild-type prostate adenocarcinomas using the Mann–Whitney test statistics (Supplementary Fig. 2, *P*-value cutoff set at 1%).

### Trigger score and prostate tissue specificity

RNA-seq data of 183 individuals from 1000 Genomes Project with available FASTA files and matched genotype data were aligned to the reference genome hg19 using STAR aligner^31^ and logarithm transformed (two based) RPKM+1 of each gene (UCSC knownGenes) were computed using mrfQuantifier^32^ and were quintile normalized.

For each of the top selected trigger score variants (*N* = 300), we measured the trigger score prostate specificity by comparing the score computed from the benign prostate tissue samples and the score computed from the 1000 Genomes Project samples. We performed 100 random sampling of 63 individuals from the 1000 Genomes Project samples set (to mimic the prostate tissue sample size) and computed the trigger score for all top 300 variants. We then annotated a variant as non-global, if no positive score was observed across the 100 experiments; a global trigger score was annotated if at least one experiment provided a positive score. A total of 69 (23%) variants showed non-global scores, where the score was positive only in the prostate tissue dataset (see Supplementary Data 3). No association between variants MAF and global or non-global annotations was detected.

### Genotype/phenotype association analysis

Genotype/phenotype association analysis was performed on the top selected trigger score variants (*N* = 300), after excluding variants with genotyping call rate <85% (*N* = 423) (Supplementary Data 5). Logistic regression analysis was used to test genotype/phenotype associations and was performed using PLINK 1.07^33^ considering allelic, dominant and recessive models. Dominant and recessive models were tested for the minor allele. Association analyses were performed applying age and PSA correction as available. In order to minimize genotype/phenotype-FDR, we computed multiple rounds of discovery and validation by partitioning the whole data set in two subsets two hundred times for each somatic phenotype (lesions in *SPOP*, *TMPRSS2-ERG*, and *FOXA1*) by preserving the lesion incidence in each subset. Specifically, in each partition the genotype/phenotype association was tested for the top selected variants applying corrected *P*-value (FDR) cutoff at 20% and *P*-value cutoff at 5% for discovery and validation, respectively. A genotype/phenotype association was considered only if both thresholds were met.

Variant 7p14.3 genotypes were tested for association with total number of tumor SNVs and with tumor genomic burden in 427 and 474 patients, respectively, (data availability as per cBioPortal), using Mann–Whitney statistics. The tumor genomic burden was measured as the fraction of the genome with absolute log2(ratio) of tumor over normal above conventionally used threshold (0.15) (Supplementary Fig. 1).

### Protein–protein interaction analysis

To characterize the PPI network of the transcriptome fraction modulated by the study variant, we first built a reference PPI network by merging information of five databases: BioGRID http://thebiogrid.org/(Release 3.2); HPRD http://www.hprd.org/ (Release 9 20100413); IntAct http://www.ebi.ac.uk/intact/ (Release 20150120); MINT http://mint.bio.uniroma2.it/mint/ (Release 20130326); STRING http://string-db.org/ (Release 9).

Interactions between nodes that represent human proteins and with a confidence score greater than 0.7 were retained (all HPRD interactions were included because no score measure is associated to protein–protein interactions in that database). The resulting network contains 263,369 interactions and 16,002 human proteins. The PPI network involving 7p14.3 variant-associated genes was built from the reference PPI network and composed of 953 genes and 1755 interactions.

To determine how likely is that the fraction of the transcriptome modulated by a variant reflects in a PPI network with a connected component comparable to the 7p14.3 variant network component, which is made of 552 genes and 1717 interactions (Supplementary Fig. 3), we built three distributions: (i) for each functional variant considered in the study (i.e., functional variants in active enhancers), the relative proportion of the biggest connected component present in the corresponding induced PPI network was calculated and a reference distribution was built; (ii) 10,000 random variants along the genome were selected (among all variants available in the Affymetrix SNP 6.0 platform) and the relative proportion of the biggest connected component present in corresponding induced PPI network was calculated for each variant to build a reference distribution; and (iii) using all genes from the reference PPI network (*N* = 16,002), 10,000 random sets of size 953 were generated and the reference distribution of the relative proportions of the biggest connected component present in the induced network was built. *P*-values were then computed for 7p14.3 variant-induced network using the three computed reference distributions (see Supplementary Fig. 4). Graphical visualization of PPI networks was performed using both igraph library^34^ of R programming language and Cytoscape tool^35^.

Pathway enrichment analysis was performed for the genes in the 7p14.3 variant PPI connected component (*N* = 552) on the REACTOME pathway database^36^ using ReactomePA R library^37^ (version 1.14.4). Oncogenes and tumor suppressors (*N* = 57) targets enrichment was performed using permutation statistics based on target genes information from TRRUST database^15^.

### TF DNA-binding sites analysis

We collected 4920 unique TF DNA-binding sites (TFBSs) consensus motifs from Jaspar^38^, HOMER^39^ and HOCOMOCO^40^ public databases and from TRANSFAC Professional database^41^. We run the transcription element search system (TESS)^42^ on the variant locus against the compiled consensus motif collection to search for TFBSs. Both ancestral and minor alleles for 7p14.3 variant were separately tested considering 30 bp flanking regions (length of collected consensus motifs ranges between 5 and 30). For each tested TFBS consensus motif, the TESS tool provides a set of log-likelihood-ratio-based scores. Specifically, we used the score La, which represent the log-odds ratio of the match, and the score Lm, which represents the maximum possible log-odds ratio for a match from the given TFBS consensus motif. To select high confident results we restricted the TFBS matches to La scores that are statistically significant (*P* < 0.001) if compared with a distribution of score matches computed from random regions of the genome. Briefly, given a consensus motif of length *N* > 6, we selected 10,000 random regions of length *N* (preserving uniqueness of the selected regions sequence) across the human genome; for *N* < = 6 all possible sequences of length *N* are considered (e.g., the number of possible sequence of length 5 is 4^5^ = 1024). Then, of those TFBS that have significant match (*N* = 32) in at least one of the two tested conditions (variant locus with ancestral or minor allele), only those with La/Lm score greater than 0.75 in at least one of the two conditions are finally retained (*N* = 7). The list of TFBS consensus motifs across the variant is reported in Supplementary Data 8. TFBS consensus motif search across a larger genomic sequence around the variant to specifically identify other CEBPB and AR motifs and possible CEBPB co-factors (c-MYC, MAFB, ONECUT1, HNF1A, E2Fs, KFLs)^17^ was performed with the less stringent 0.005 *P*-value for La score filtering and the less stringent 0.6 La/Lm score cutoff; see Supplementary Data 8.

### Linkage disequilibrium analysis

In all, 1000 Genome Project genotype data of 1899 individuals from four populations (EUR, AFR, SAS, and EAS) was considered. 54,892 variants within 1 Mbp flanking regions around rs1376350 were analyzed. *R^2* and *D′* coefficients of linkage disequilibrium between variant rs1376350 and all other variants were computed using library genetics of R programming language (http://CRAN.R-project.org/package=genetics). Variants were annotated regarding their presence on the Affymetrix SNP 6.0 platform. Variants with *R^2* > 0.10 were reported in Supplementary Data 6.

### Cell lines

PC-3 (prostate cancer metastatic site derived, bone) and LNCaP (prostate cancer metastatic site derived, lymph node) cells were maintained in RPMI medium (Gibco, Life Technologies, Milan, Italy), supplied with 10% FBS, 100 units/ml penicillin, 100 µg/ml streptomycin, and 2 mM l-Glutamine, at 37 °C with 5% CO_2_. Sex hormone depletion (androgens and estrogens), prior to DHT (Sigma-Aldrich, Milan, Italy) treatments, was achieved by growing the cells in medium without phenol red (Euroclone, Celbio, Milan, Italy), supplemented with 10% charcoal/dextran treated FBS (Hyclone, Celbio, Milan, Italy) for 48 h. The cell lines were purchased from ATCC (American Type Culture Collection, LGC Standards). PC-3 and LNCaP are GG at rs1376350 (GSM888588, GSM888346).

### Plasmids and luciferase assay

The genomic sequence spanning 7p14.3 variant was generated from PC-3 genomic DNA using primer pairs as detailed in Supplementary Data 10. For the identification of enhancer activity the fragment was cloned in pGL4.26 (Promega) in which firefly luciferase is driven by a minimal promoter. The PCR fragment was cloned upstream of the firefly luciferase gene using Kpn1 and XhoI restriction enzymes. Constructs harboring the alternative allele for the study variant (adenine) was created with the GeneArt Site-Directed Mutagenesis System (Invitrogen, Life Technologies) according to the manufacturer’s instructions. The correct insertion of the genomic sequence was confirmed by restriction enzyme digestion and sequence analysis (Eurofins genomics). All plasmids were purified from DH5α *E. coli* bacterial cells using the PureYield Plasmid Midiprep system protocol (Promega). The day before transfection, PC-3 cells (8 × 10^4^ cells) were seeded in 24-well plates. Cells were transfected using TransIT-LT1 reagent (Mirus, TemaRicerca) with pGL4.26-derived vector (350 ng). pRL-SV40 vector (50 ng) (Promega) was used to normalize the transfections efficiency. Construct harboring cytosine allele showed efficiency levels consistent with reference allele. In a parallel experiment, PC-3 cells were cotransfected with pGL4.26-derived vector and pCMV-AR24Q expression vector and/or pCMV6_CEBPB (100 ng, to over-express AR or CEBPB) and treated with 100 nM DHT for at least 16 h. CEBPB or AR silencing was performed by transfection of PC-3 or LNCaP cells with siRNA against CEBPB or AR (20 nM) (FlexiTubeGeneSolution for CEBPB or AR, Qiagen) and Hiperfect transfection reagent (Qiagen) or Lipofectamine 2000 (Thermo Fisher Scientific), respectively. AllStars Hs Cell Death siRNA and AllStars Negative Control siRNA (Qiagen) were used as positive and negative control (Supplementary Fig. 15). Forty-eight hours after over-expression or 72 h after silencing, cells were lysed using Passive Lysis Buffer 1X (Promega) and Firefly and Renilla luciferase activities were measured with Dual-Luciferase Reporter Assay (Promega) using the Infinite M200 multi-plate reader (Tecan).

### ChIP assay

PC-3 cells were maintained into 150 mm Petri dishes in RPMI medium without phenol red, supplemented with 10% charcoal/dextran treated FBS. Two days after, as PC-3 cells do not express AR^8^, they were transfected either with pCMV-AR24Q expression vector or with the pCMV-NeoBam empty vector. Then, cells were treated with EtOH or DHT (100 nM) and after 16 h of treatment, cells were subjected to ChIP with an anti-AR antibody (3 μg, 17-10489 ChIPAb + androgen receptor Assay Kit, Millipore), anti-CEBPB antibody (3 μg, 18F8 Abcam), anti c-Myc antibody (3 μg, N262 Santa Cruz Biotechnology) or a normal IgG (3 μg, CS200581), using the MagnaChIP HiSens ChromatIPKit (17-10461 Upstate, Millipore) as previously described^8^. Briefly, the experiment procedure includes chromatin crosslinking with formaldehyde, chromatin shearing for 45 cycles of 30 s ON/30 s OFF with the Bioruptor Pico (Diagenode), protein–DNA complex immunoprecipitation and reverse crosslinking with protease K. Precipitated DNA was analyzed by real-time qPCR with KAPA SYBR FAST Universal 2X qPCR Master Mix (Kapa Biosystems, Resnova) using the CFX384 or CFX96 Detection Systems (BioRad). KLK3 enhancer region, IL-6 promoter region and NPM1 intron 1 were used as positive control of AR, CEBPB and c-Myc, respectively; a desertic region in chr12 as per ENCODE annotations (hg19, chr12:17456963-17457066) was used as negative control. The region of interest surrounding the 7p14.3 variant was amplified as well (Supplementary Data 10). AR, CEBPB or c-Myc specific recruitment was calculated as enrichment respect to the IgG according to the ΔCt method.

### CRISPR-Cas9 7p14.3 deleted locus in PC-3 cells

Single guide RNA oligos (sgRNAs) to induce 7p14.3 locus deletion were selected using the GPP Web Portal (http://portals.broadinstitute.org/gpp/public/) that ranks candidates according to their predicted on-target and off-target activity (Supplementary Data 9). Selected sequences were ligated into pUC19 that contains U6 promoter-driven cassette, derived from px330 (Addgene 42230). Plasmid eSpCas9(1.1)-2A-Puromycin, derived from Addgene 71814, were generated through the addition of nucleotides encoding 2 A peptide and Puromycin resistance^43^. To induce genomic deletions four pairs of sgRNA (listed in Supplementary Data 9) vectors were cotransfected with Cas9 expression vector. In a 6 well plate, 3 × 10^5^ PC-3 cells/well were seeded and after 24 h were transfected with 1.5 µg of eSpCas9(1.1)-2A-Puromycin plasmid and 250 ng of sgRNA plasmid using FuGENE HD (Promega E2311). Three days after transfection, cells were selected for 7 days with 2 µg/ml Puromycin (Sigma-Aldrich P8833) in order to prioritize transfected cells only. A representative amount of cells was used for DNA extraction and the remaining cells were re-plated and cultured for RNAseq experiments. Genomic DNA was extracted following the procedures of NucleoSpinTissue kit (Macherey-Nagel). To evaluate the editing, 25 µl PCR reaction was performed using 12.5 µl Platinum SuperFi Green PCR Master Mix (2X), 50 ngDNA template and 0.5 µM forward and reverse primers (Supplementary Data 10). Predicted PCR bands (Supplementary Fig. 16), verified by sequencing, are listed in Supplementary Data 9. Based on editing efficiency, combinations A and B were selected for downstream experiments (deleted segments of length 625 bp and 731 bp, respectively).

### RNA-seq experiments

PC-3 cells (7p14.3 deleted and not deleted cells) were seeded in 24-well plate, transfected with pCMV-AR24Q expression vector using FuGENE HD (Promega E2311) and then treated with DHT for at least 16 h. CEBPB silencing was performed by transfection of PC-3 cells with siRNA against CEBPB (20 nM) (FlexiTubeGeneSolution, Qiagen) and Lipofectamine RNAiMAX Transfection Reagent (Invitrogen, 13778150). Total RNA was extracted using the RNeasy kit (Qiagen) according to the manufacturer’s instructions. The RNA integrity number (RIN) was quantify on the Agilent 2100 bioanalyzer. cDNA libraries were prepared with TruSeq stranded mRNA library prep Kit (RS-122-2101, Illumina) using 500 ng of total RNA. Single end (100 bp) sequencing was performed on a HiSeq 2500 (Illumina). FASTA files were aligned to the reference genome hg19 using STAR aligner^31^ and logarithm transformed (two based) RPKM+1 of each gene (UCSC knownGenes) were computed using mrfQuantifier^32^ and were quintile normalized. The resulting expression data was used to identify variation in gene expression in edited vs. control cells in all treatment conditions (16 sample’s combinations) and across treatment conditions for both edited and control cells separately (12 sample’s combinations). For each combination, we considered transcripts with RPKM greater than 1 in at least one of the two samples (values below 1 are set to 1) and selected only those with absolute log2(ratio) equal or greater than 1. Concordance of deregulation in cells edited with A and B sgRNAs combinations is shown in Supplementary Fig. 17 and deregulated transcripts across experimental conditions in 731 bp edited cells (combination B) are reported in Supplementary Data 11. Evidence of deregulation enrichment was tested by comparing the abundance of deregulation in combinations B edited vs. control cells and in control vs. control or edited vs. edited cells (Supplementary Fig. 11). Hi-C data previously generated in RWPE1 prostate cells^18^ was queried to test evidence of deregulation at chromosome 7 in correspondence of 7p14.3 Hi-C links (Fig. 3a, b). Hi-C links are defined as genomic regions with normalized Hi-C signal above the 90th percentile of the overall intra-chromosomal 7p14.3 normalized Hi-C signal distribution (Supplementary Fig. 18).

### Real-time qPCR

PC-3 cells were seeded in 6-well plate. Total RNA was extracted using the RNeasy kit (Qiagen) according to the manufacturer’s instructions. Two-hundred nanogram of total RNA was retro-transcripted into cDNA using the Revert Aid First Strand cDNA Synthesis Kit (ThermoFisher Scientific). Then, qPCR reactions in real-time were performed using KAPA SYBR FAST Universal 2X qPCR Master Mix (Kapa Biosystems, Resnova) using the CFX384 or CFX96 Detection Systems (BioRad). Analysis of relative mRNA expression was performed using the ΔΔCt method with *GAPDH* (glyceraldehyde 3-phosphate dehydrogenase) as reference genes (primers sequences in Supplementary Data 10). RNA-seq validation of selected transcripts (Supplementary Data 10) was performed with qPCR including RAB1B, BAP1, and BCAP31 as negative controls. As control cells, we used PC-3 transfected with pSpCas9(1.1)^43^, sgRNA_scramble, and pGL4.14. After 72 h from transfection, cells were selected with Puromycin for 4 days and harvest for 4 weeks.

### Western blot

Proteins were extracted from PC-3 cells with ice-cold RIPA (Radio Immuno-Precipitation Assay) lysis buffer supplemented with protease inhibitor cocktail (11873580001, Roche), then homogenized in a dounce homogenizer for 1 h and centrifuged at 13,400 rcf at 4 °C for 10 min. The supernatants were collected and boiled with 6x sample buffer at 95 °C for 5 min. The samples were separated by sodium dodecyl sulfate polyacrylamide gel electrophoresis and transferred to nitrocellulose membranes using wet transfer or the semi-dry iBlot Transfer System (Invitrogen, Life Technologies). The membranes were blocked with 5% non-fat dry milk in PBS-T for 1 h, then incubated with either anti-CEBPB (Abcam 18F8, 1:1000 dilution), anti-AR (Cell Signaling 5153 S, 1:1000 dilution), anti-c-Myc (Santa Cruz N262,1:1000), anti-GAPDH (Santa Cruz Biotechnology, sc-322330 dilution) or anti-beta-Tubulin (Santa Cruz Biotechnology 3F3-G2, 1:8000 dilution) antibody in 1% non-fat dry milk in PBS-T overnight at 4 °C. Membranes were then incubated with secondary goat anti-mouse (Santa Cruz Biotechnology A9044, 1:10000) or goat anti-rabbit antibodies (Sigma-Aldrich A9169, 1:12,000) for 1 h at room temperature. Detection was achieved using the ECL Select detection reagent (Amersham, GE Health Care) with the ChemiDoc XRS + System (BioRad) (Supplementary Figs. 19 and 20).

### Data availability

RNA-seq data of control and edited PC-3 cells have been deposited at BioProject database under the accession code PRJNA381797. All other remaining data are available within the article and Supplementary Files, or available from the authors upon request.

## Electronic supplementary material

Supplementary Information

Supplementary Data 1

Supplementary Data 2

Supplementary Data 3

Supplementary Data 4

Supplementary Data 5

Supplementary Data 6

Supplementary Data 7

Supplementary Data 8

Supplementary Data 9

Supplementary Data 10

Supplementary Data 11
