# Supplementary material for: Inherited determinants of early recurrent somatic mutations in prostate cancer
Source: Nat Commun. 2017 Jun 29;8:48. doi: 10.1038/s41467-017-00046-0 (PMC5491529; doi:10.1038/s41467-017-00046-0)
Supplement: Supplementary file 1 — Supplementary Information [file 41467_2017_46_MOESM1_ESM.pdf]

Supplementary Figures

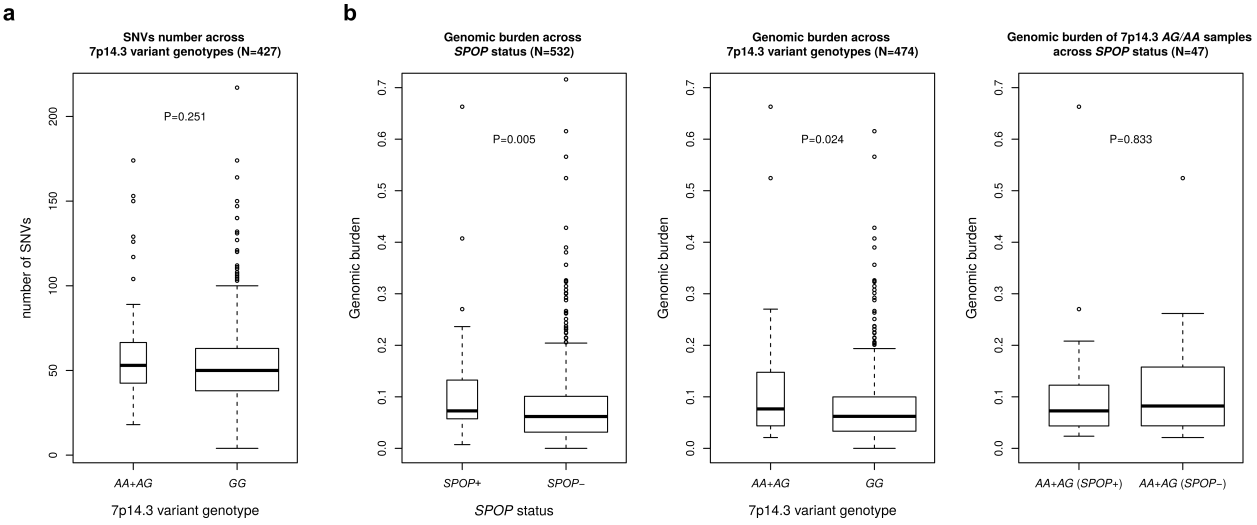

Supplementary Figure 1: SNV burden and genomic tumor burden across 7p14.3 variant genotypes and SPOP mutation. a) Total number of somatic SNVs versus 7p14.3 variant genotype in a collection of prostate cancer men. **b)** Association of *SPOP* mutation status versus somatic genomic tumor burden (left), 7p14.3 variant versus somatic genomic tumor burden (middle), and 7p14.3 variant minor allele and somatic genomic tumor burden across *SPOP* mutation status (right). P-values are computed using Mann-Whitney test statistics.

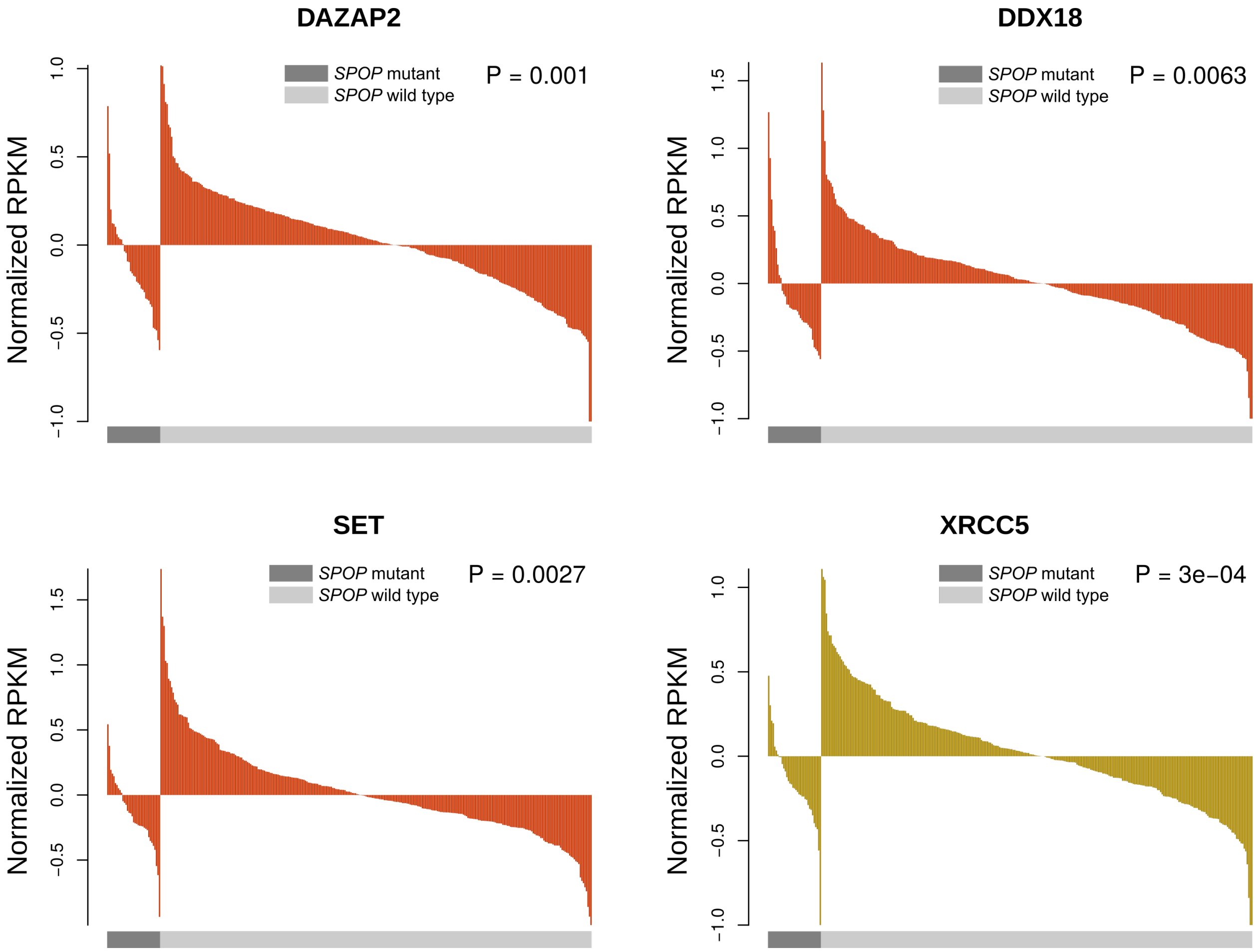

Supplementary Figure 2: Differential expression across *SPOP* mutated and *SPOP* wild type prostate tumors. DNA repair and hormone regulated genes associated to 7p14.3 variant with significant (Mann-Whitney test statistics) differential expression across *SPOP* mutant and *SPOP* wild type prostate adenocarcinomas. Normalized RPKM values are computed by dividing RPKM values by the mean value across all samples and centering the signal with the mean at 0. Samples are sorted for decreasing level of transcript within *SPOP* mutant and *SPOP* wild type groups.

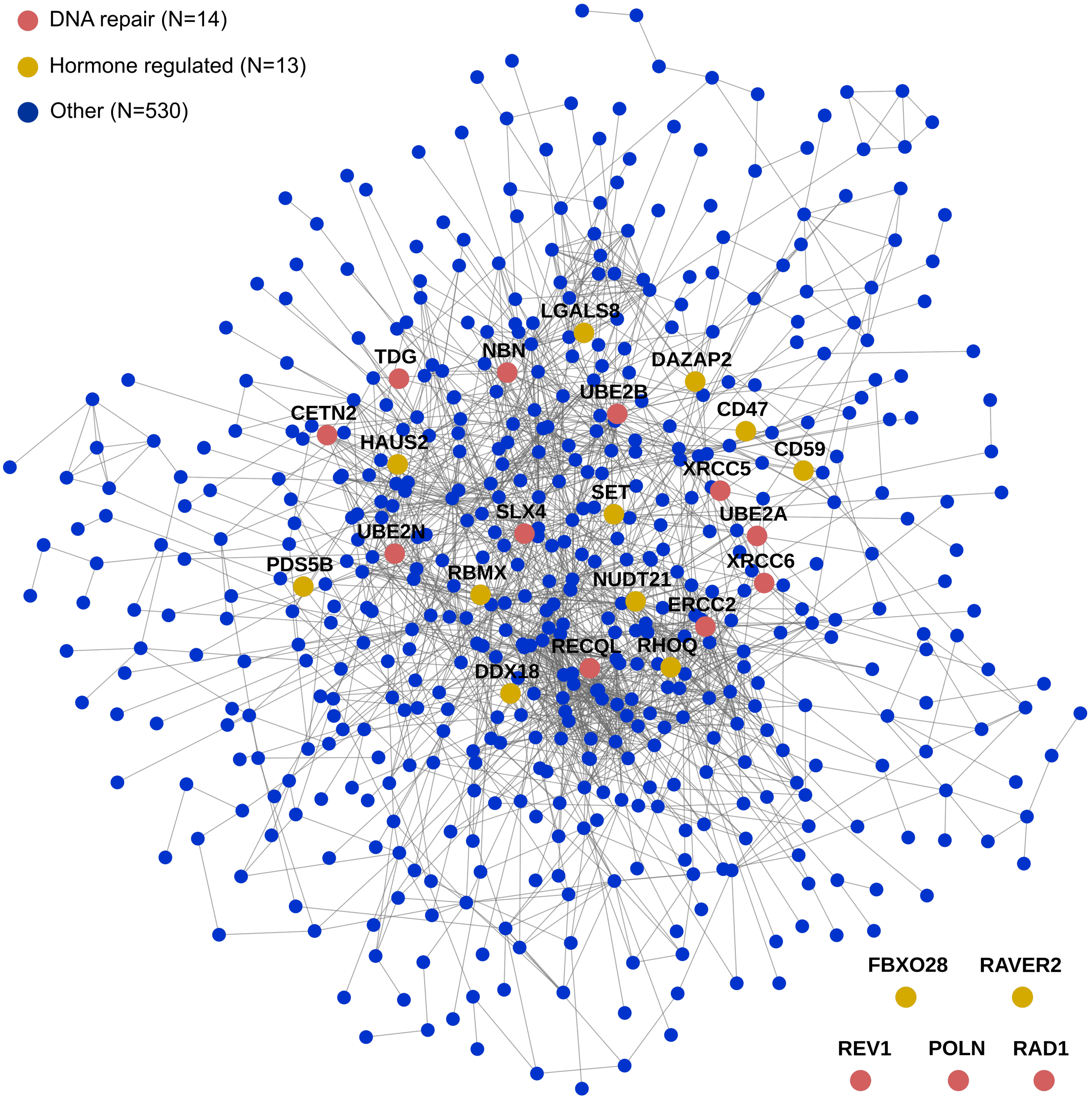

Supplementary Figure 3: PPI network analysis. Maximal connected component of 7p14.3 induced PPI network with DNA repair and hormone regulated genes. Two genes contributing to the high trigger score are not included in the PPI databases.

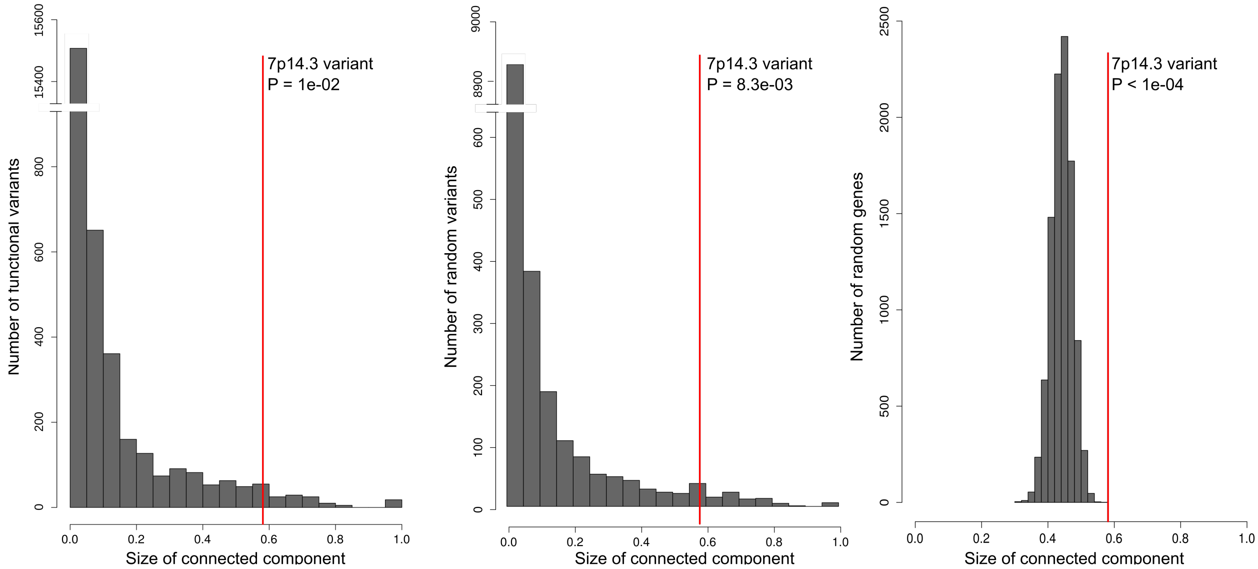

Supplementary Figure 4: Significance analysis of the 7p14.3 PPI network connected component size. Distribution of the relative proportion of the largest connected component present in the PPI networks corresponding to functional variants considered in the study (left); Distribution of the relative proportion of the largest connected component present in the PPI networks corresponding to 10,000 random variants selected along the genome (middle); Distribution of the relative proportion of the largest connected component present in the PPI networks corresponding to 10,000 networks constructed from 953 random genes (right). P-values are computed using resampling statistics.

a

AR consensus (M00962 TRANSFAC)

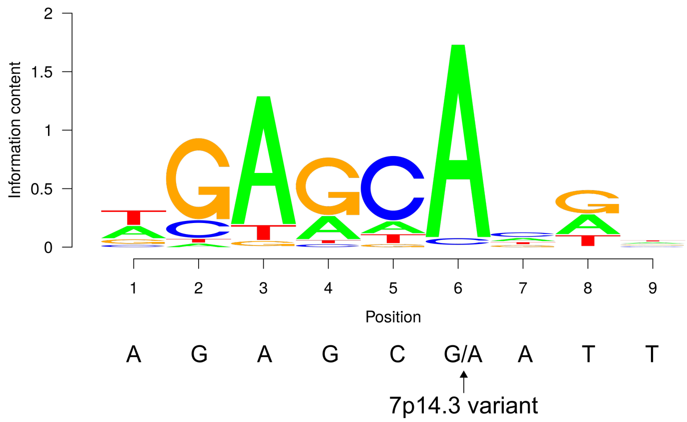

	ancestral allele G	minor allele A
La score	1.87	8.74
La/*Lm* score	0.18	0.82
p-value	0.08	0.0009

b

CEBP family consensus (M00770 TRANSFAC)

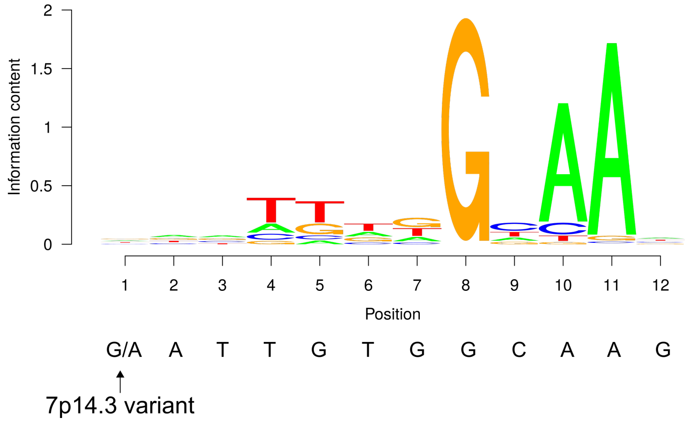

	ancestral allele G	minor allele A
La score	8.97	8.97
La/*Lm* score	0.77	0.77
p-value	0.0002	0.0002

Supplementary Figure 5: Consensus recognition site of AR and CEBPB. a) AR consensus motif found at 7p14.3 locus**. b)** CEBP family consensus motif found at 7p14.3 locus. For each consensus motif, matching scores *La* and *La/Lm* and matching p-values (computed using permutation test statistics) are reported both for the ancestral and the minor allele.

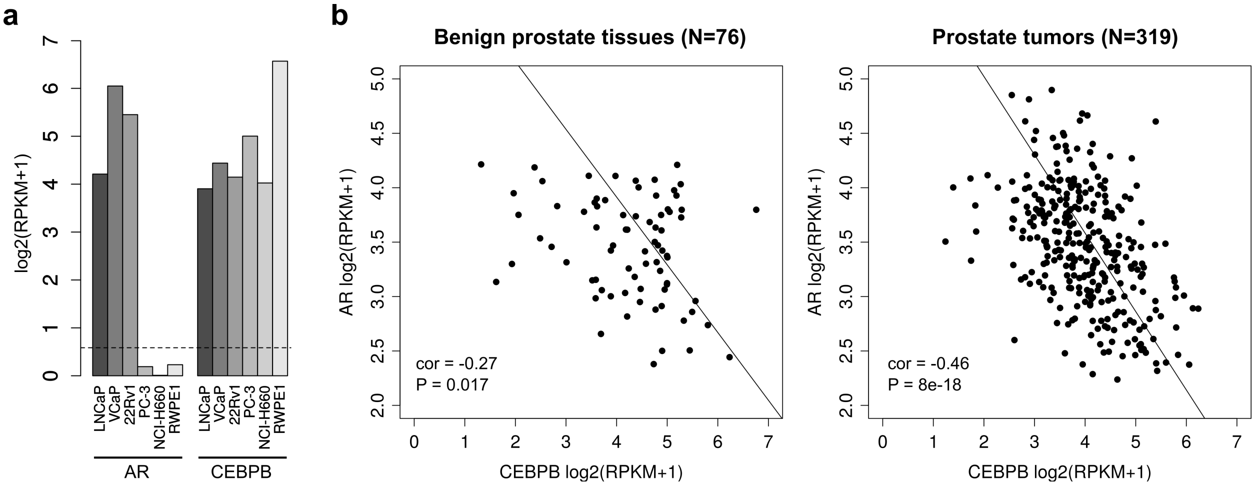

Supplementary Figure 6: RNA-seq levels of AR and CEBPB. a) Normalized RPKM transcript levels of AR and CEBPB are shown for a series of prostate cell lines profiled with RNA-seq experiments. **b)** Normalized RPKM transcript levels of AR versus CEBPB in study cohorts. Correlations and corresponding p-values were measured with Pearson correlation coefficient from benign prostate tissues (left) and prostate tumors (right) RNA-seq experiments. Black lines represent regression lines.

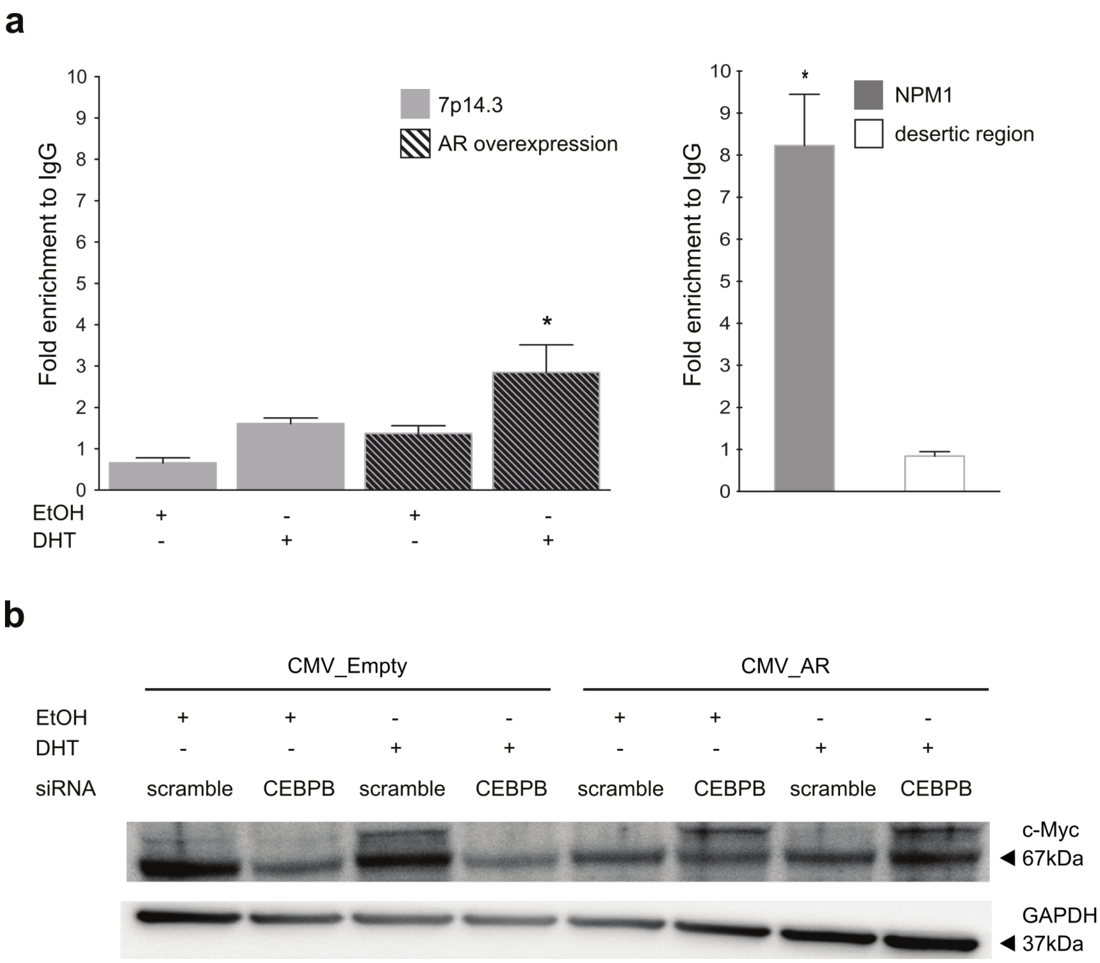

Supplementary Figure 7: c-Myc recruitment at the 7p14.3 region. a) PC-3 cells were transfected with pCMV_Empty (solid bars) or pCMV_AR (dashed bars) vectors, and c-Myc chromatin binding at 7p14.3 locus was evaluated. Occupancy level at NPM1 Intron 1 was used as positive control. c-Myc recruitment at region of interest is observed upon AR overexpression and DHT treatment (fold enrichment to IgG) (mean ± s.d. of two biological replicates). **b)** Protein levels of c-Myc were evaluated through western blot analysis. Upon CEBPB silencing and/or AR overexpression, the protein level decreased. Uncropped blot image in Supplementary Figure 20. *p<0.05, Student's t-test.

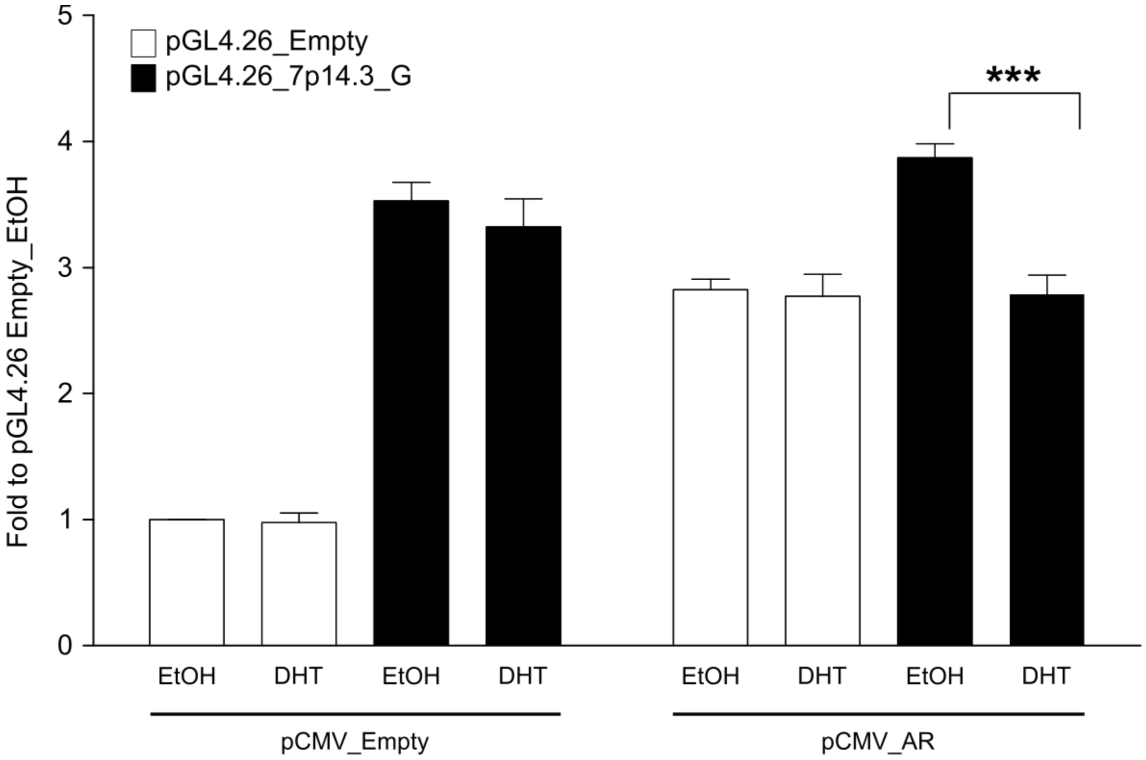

Supplementary Figure 8: Responsiveness at 7p14.3 cloned regulatory region in PC-3 cells. PC-3 cells were co-transfected with pGL4.26 empty vector (white) or containing the locus 7p14.3 (G allele, black), along with pCMV_Empty or pCMV_AR. The enhancer activity was lower upon AR overexpression and DHT treatment (mean ± s.d. of two biological replicates). *p<0.05, **p<0.01, ***p<0.005, Student's t-test.

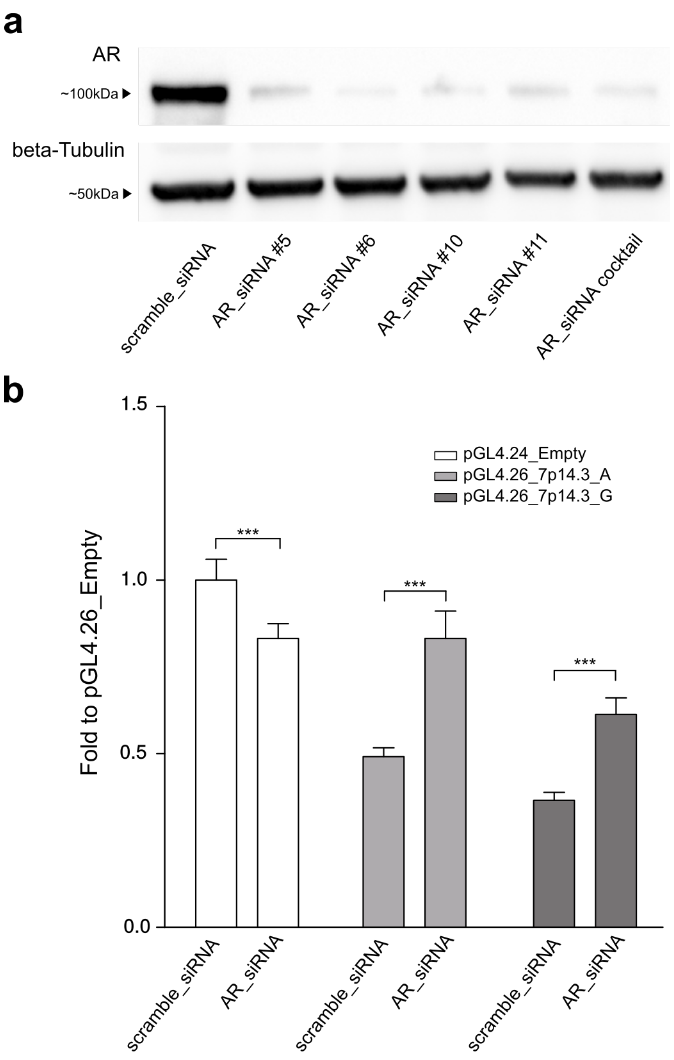

Supplementary Figure 9: Responsiveness at 7p14.3 cloned regulatory region in LNCaP. **a)** The amount of AR protein levels were evaluated through western blot analysis. In AR knock-down cells a strong consistent reduction of the AR protein was observed. **b)** LNCaP cells were transfected with siRNA against AR or with scrambled siRNA along with the pGL4.26 reporter vectors. The repressor activity was lower upon AR silencing (mean ± s.d. of three biological replicates). *$p<0.05$, **$p<0.01$, ***$p<0.005$, Student's t-test

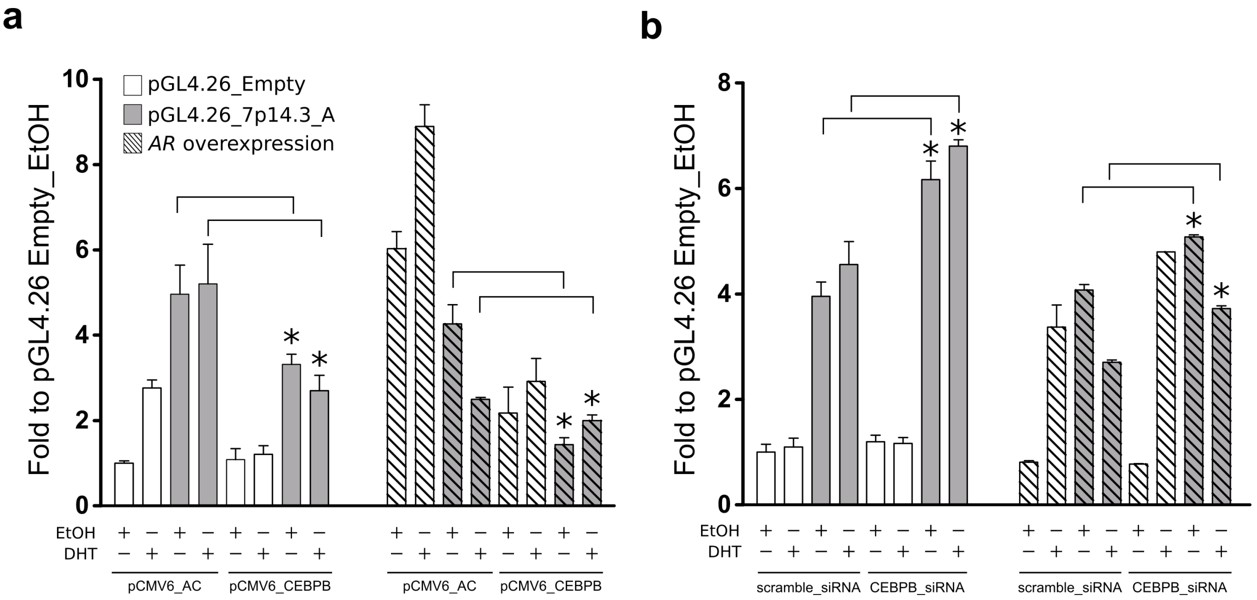

Supplementary Figure 10: Co-transfection in PC-3 of the reporter with cloned variant A allele. PC-3 cells were co-transfected (see **Figure 2c-d**) in order to overexpress **(a)** or knock-down **(b)** CEBPB contextually with the pGL4.26 plasmid harboring the A allele. The luciferase activity is inhibited upon CEBPB overexpression and enhanced upon CEBPB silencing (mean ± s.d. of two biological replicates, pGL4.26_Empty are from **Figure 2c-d**). *p<0.05, **p<0.01, ***p<0.005, Student's t-test

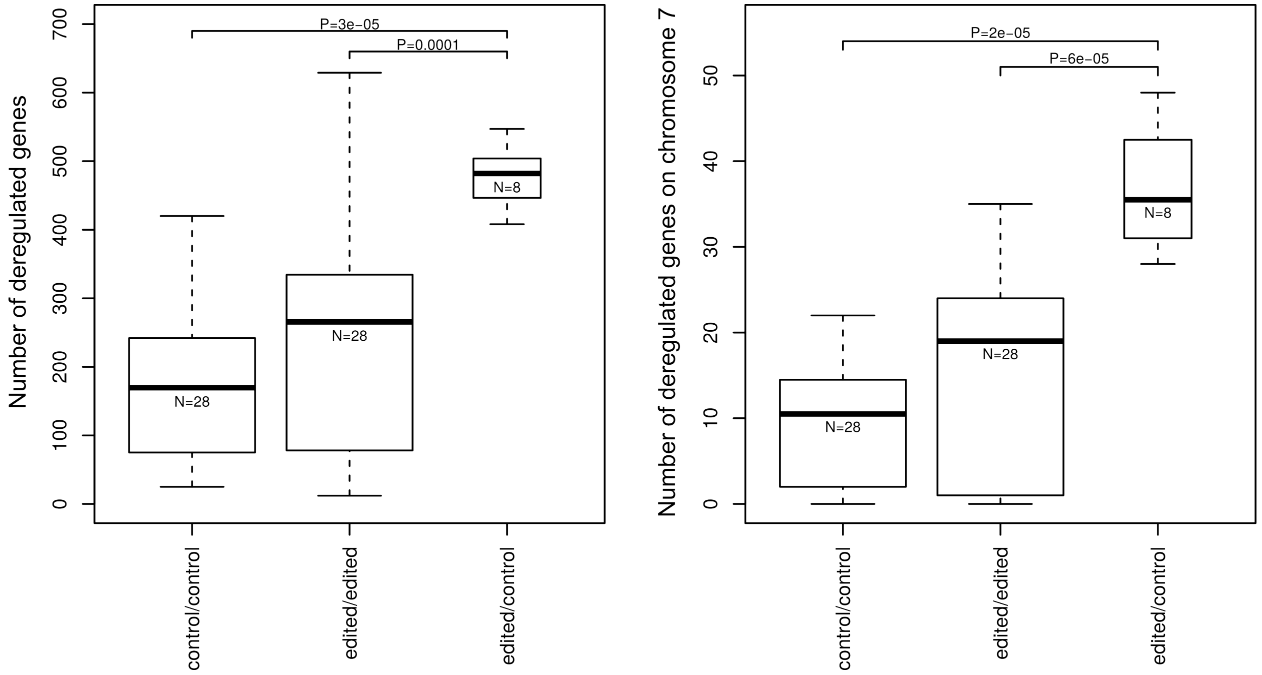

Supplementary Figure 11: Enrichment of deregulation in edited versus control cells. (Left) Total number of deregulated genes in edited versus control cells compared to control versus control cells and edited versus edited cells. (Right) Similar analysis considering only genes on chromosome 7. P-values computed using Mann-Whitney test statistics.

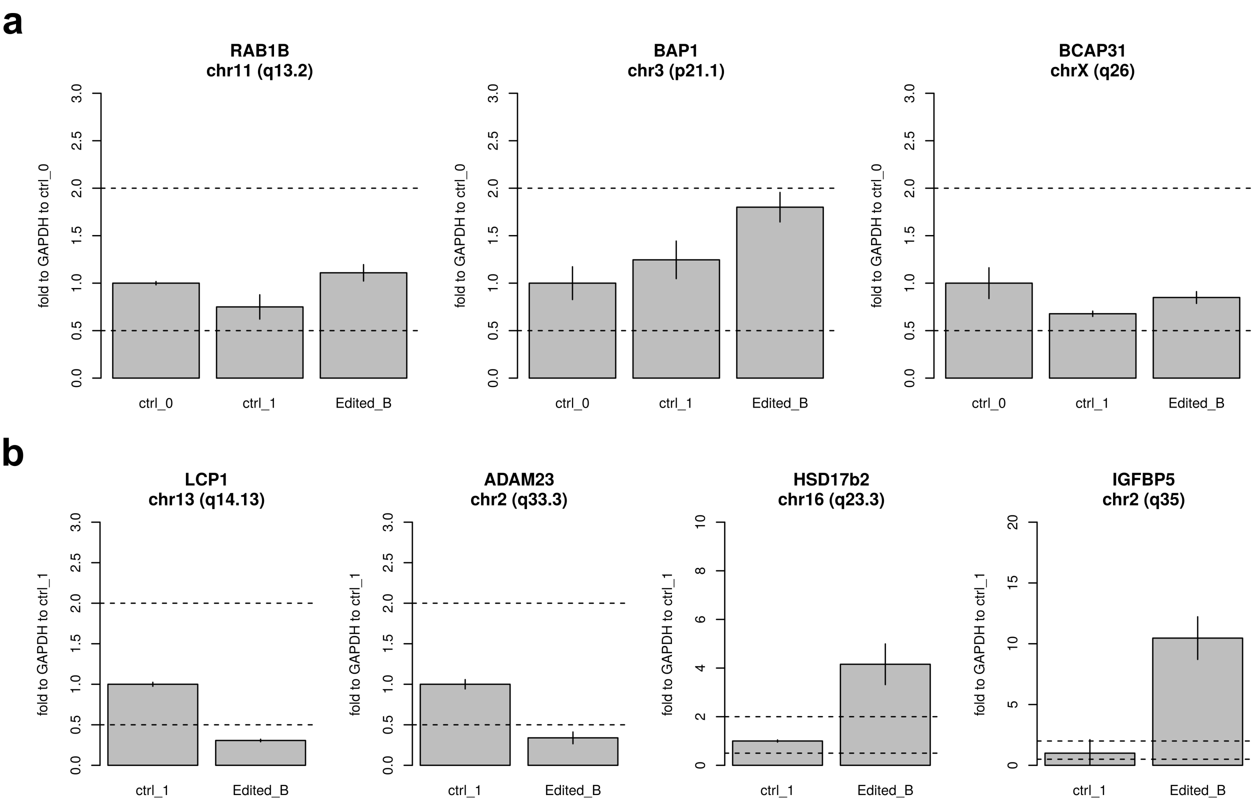

Supplementary Figure 12: Real-time validation of RNA-seq experiments. a) negative controls. **b)** Down- and up-regulated transcript levels validated through real-time were selected based on edited versus control cells. ctrl_1 are PC-3 cells transfected with pSpCas9(1.1), sgRNA_scramble, and pgl4.14 and selected with Puromycin; ctrl_0 are PC-3 cells transfected with pCMV_Empty and siRNA_scramble treated with EtOH 16h before collection. Data are represented as mean ± s.d. of three technical replicates.

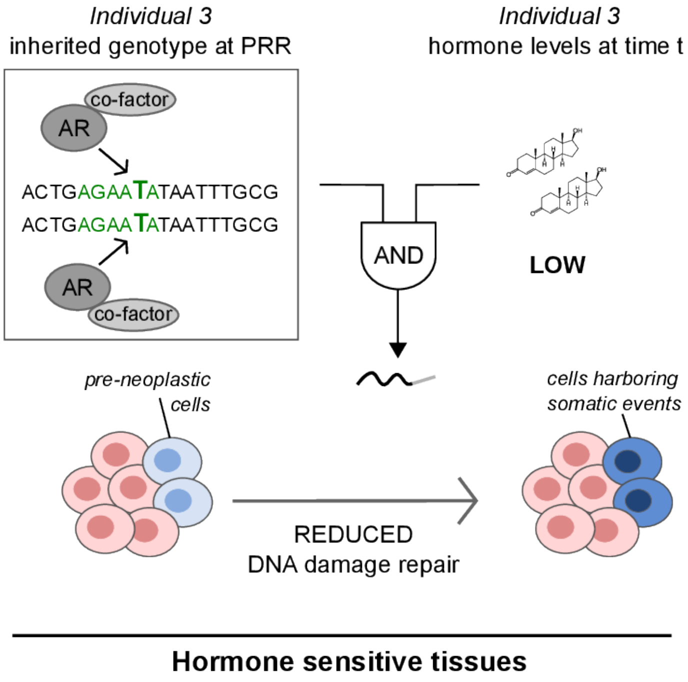

Supplementary Figure 13: **Two-variable model of genotype-environment interaction study.** Minor allele homozygous genotype of Individual 3 and low hormone levels result in reduced transcription of DNA repair genes, facilitating the emergence of early somatic events.

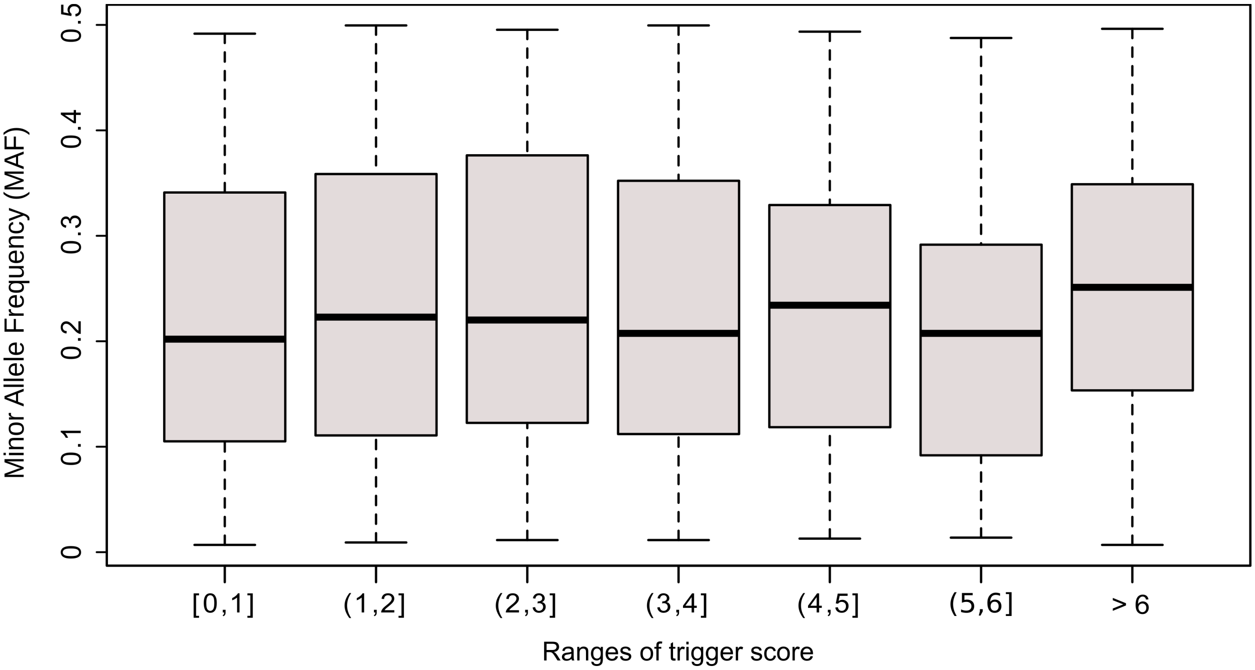

Supplementary Figure 14: Distribution of variants MAF across different trigger score ranges. Relation between variants MAF and variant related trigger score.

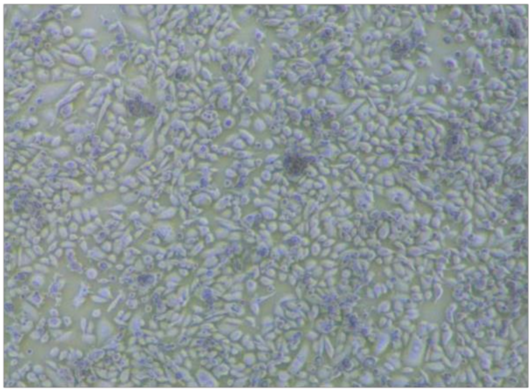

AllStars Negative Control siRNA

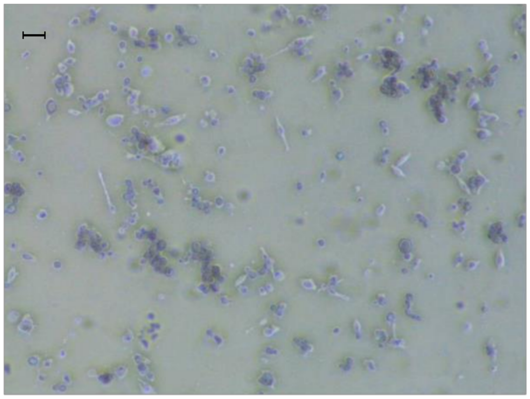

AllStars Hs Cell Death siRNA

Supplementary Figure 15: **siRNA transfection with negative and positive control.** PC-3 cells were transfected with 20nM scrambled siRNA (AllStars Negative Control siRNA) or AllStars Hs Cell Death siRNA using HiPerFect Transfection Reagent. After 72 hours, cell death was observed by light microscopy (the bar corresponds to 75 micrometers).

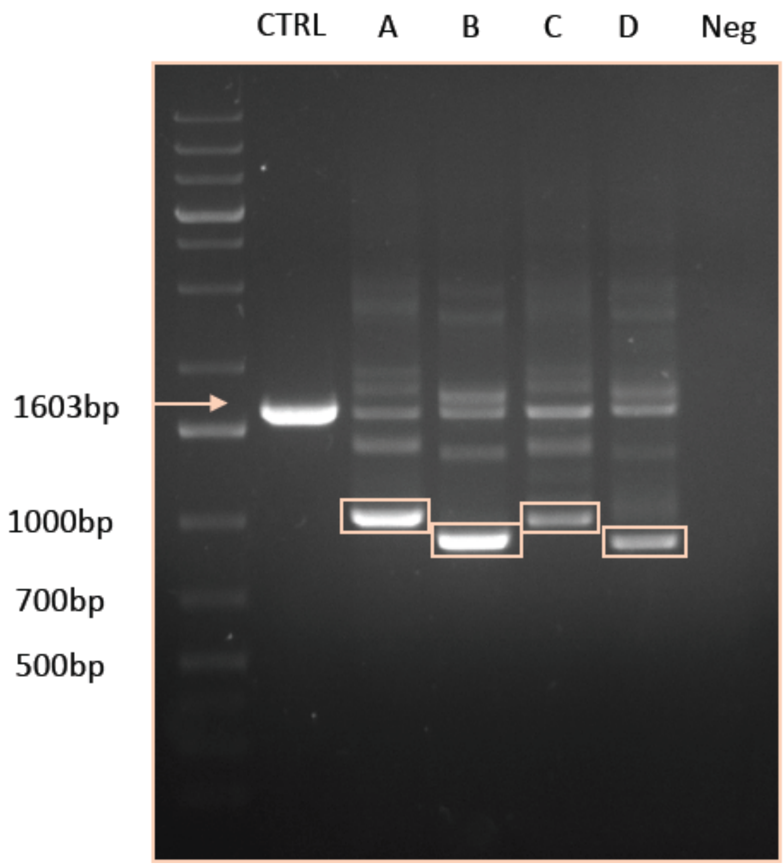

Supplementary Figure 16: PCR based screening of deletion in PC3 cells. Final PCR product of untransfected PC3 cells (CTRL) (1603bp) and of four combinations of RNA guides to obtain genomic deletion at the 7p14.3 locus were detected by agarose gel electrophoresis. Combinations A (sgRNA_up 1 and sgRNA_down 1) and B (sgRNA_up1 and sgRNA_down 2) demonstrate stronger deleted allele band with respect to combinations C (sgRNA_up 2 and sgRNA_ down 1) and D (sgRNA up_2 and down_2). The expected size of each final product of PCR representative of the deletion is reported in **Supplementary Data 9** and was verified by sequencing.

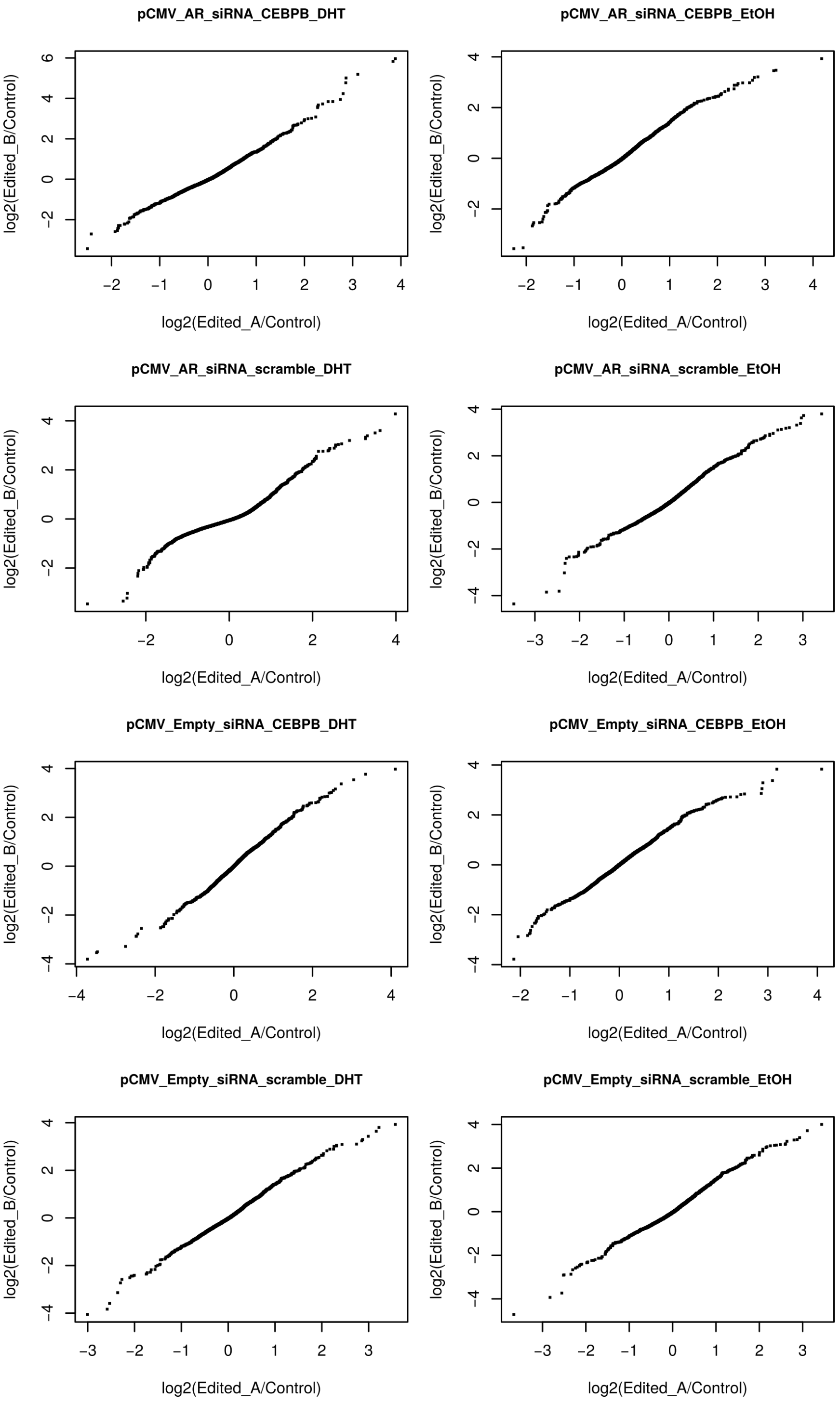

Supplementary Figure 17: Comparison of deregulation in edited cell lines. Concordance of deregulation between cells edited with A or B sgRNAs combinations across different experimental conditions.

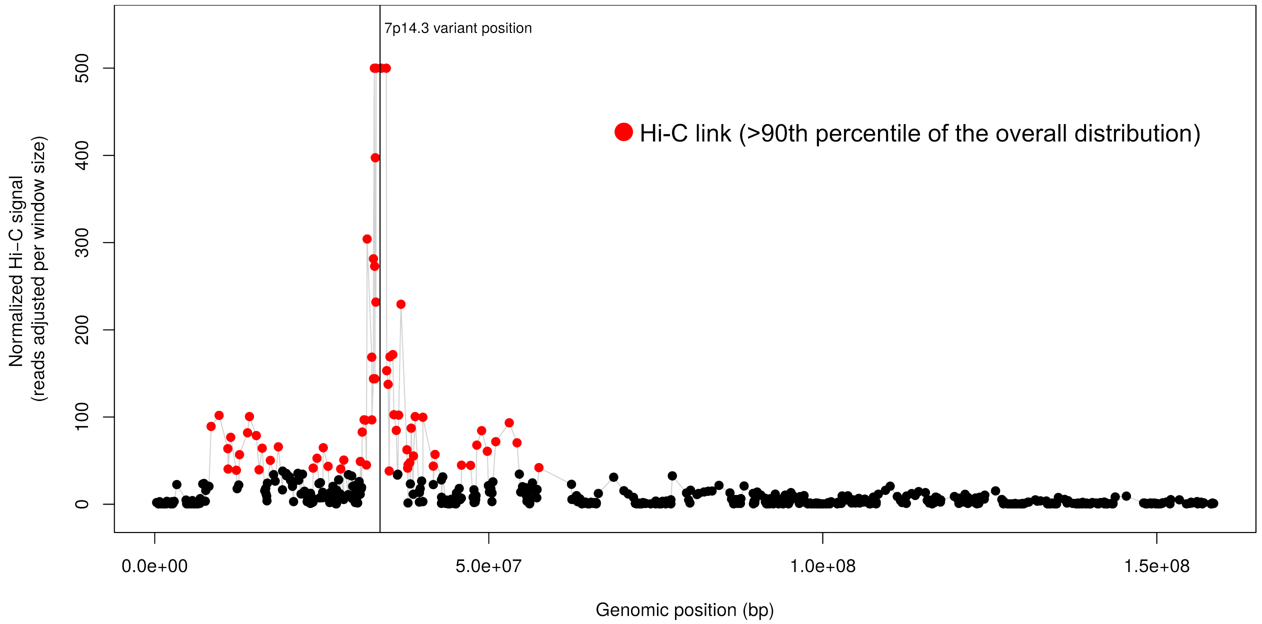

Supplementary Figure 18: 7p14.3 links detected from benign prostate cells Hi-C data. Profile of 7p14.3 contact points on chromosome 7 from RWPE1 Hi-C data. Potential Hi-C links are depicted in red.

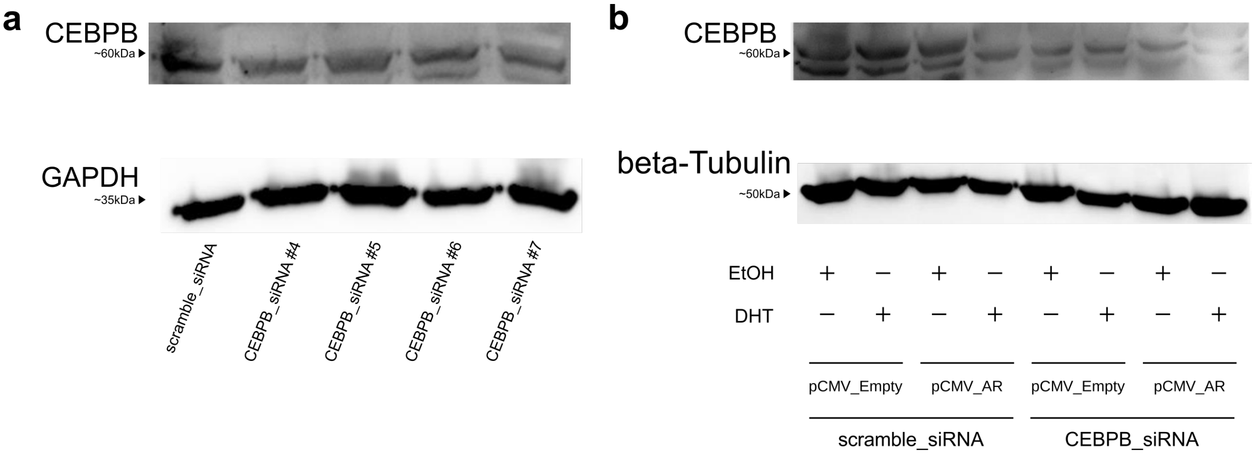

Supplementary Figure 19: CEBPB silencing in PC-3. a) PC-3 cells were transfected with siRNA against CEBPB (four different siRNAs). All four separate CEBPB siRNAs showed approximately the same effect leading to reduction of the target protein (28.16%, 26.45%, 32.77%, and 19.80% reduction compared to the control, respectively). **b)** PC-3 cells were transfected with siRNA cocktail against CEBPB or scrambled siRNA. The day after the cells were transfected with pCMV_Empty or pCMV_AR vectors and 24 hours after were treated with DHT for 16 hours. The amount of CEBPB protein levels was evaluated through western blot analysis. In CEBPB knock-down cells a strong reduction of the protein was observed. GAPDH or beta-Tubulin were used as loading control.

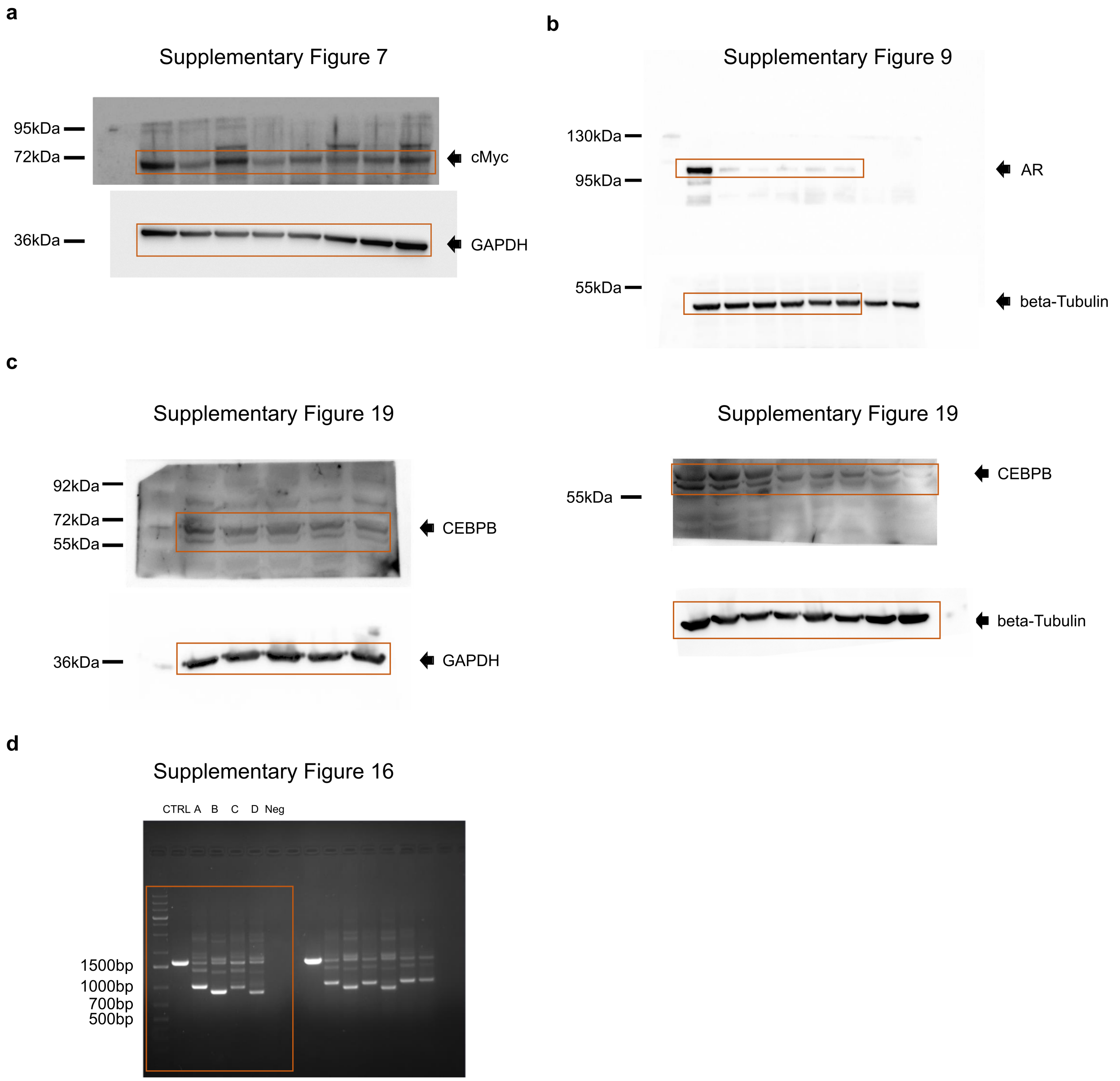

Supplementary Figure 20: **Uncropped blots and gel images. a)** Uncropped immunoblots for **Supplementary Figure 7b**. Red squares indicate the section shown in **Supplementary Figure 7b**. **b)** Uncropped immunoblots for **Supplementary Figure 9a**. Red squares indicate the section shown in **Supplementary Figure 9a**. **c)** Uncropped immunoblots for **Supplementary Figure 19a-b**. Red squares indicate the section shown in **Supplementary Figure 19a-b**. CEBPB and beta-Tubulin were developed with same samples run in two separate SDS-PAGE gels. **d)** Uncropped agarose gel image for **Supplementary Figure 16**. The red square indicates the section shown in **Supplementary Figure 16**.

Supplementary Tables

Population	1000GP MAF	Prostate Adenocarcinoma
EAS	0.118 (A=119,G=1008)	0.1 (A=2,G=20)
AMR	0.013 (A=9,G=694)	0.067 (A=2,G=30)
AFR	0.003 (A=4,G=1322)	0.012 (A=1,G=82)
EUR	0.024 (A=24,G=1006)	0.052 (A=43,G=830)
SAS	0.038 (A=37,G=978)	NA
Global	0.039 (A=193,G=5008)	0.050 (A=48,G=962)

Supplementary Table 1: Minor allele frequency of 7p14.3 variant. Minor allele frequency of 7p14.3 variant (rs1376350) in 1,000 Genome Project data (globally and across different ethnic groups) and in our Prostate Adenocarcinoma dataset. EAS = East Asians, AMR = Americans, AFR = Africans, EUR = Europeans , SAS = South Asians.
